# Distinct susceptibility of HIV vaccine vector-induced CD4 T cells to HIV infection

**DOI:** 10.1371/journal.ppat.1006888

**Published:** 2018-02-23

**Authors:** Sarah Auclair, Fengliang Liu, Qingli Niu, Wei Hou, Gavin Churchyard, Cecilia Morgan, Nicole Frahm, Sorachai Nitayaphan, Punnee Pitisuthithum, Supachai Rerks-Ngarm, Jason T. Kimata, Lynn Soong, Genoveffa Franchini, Merlin Robb, Jerome Kim, Nelson Michael, Haitao Hu

**Affiliations:** 1 Department of Microbiology and Immunology, Sealy Center for Vaccine Development and Institute for Human Infections and Immunity, University of Texas Medical Branch, Galveston, TX, United States of America; 2 School of Basic Medical Sciences, Wuhan University, Wuhan, Hubei, China; 3 The Aurum Institute, Johannesburg, South Africa; 4 Vaccine and Infectious Disease Division and the HIV Vaccine Trials Network, Fred Hutchinson Cancer Research Center, Seattle, WA, United States of America; 5 Royal Thai Army Expert, Armed Forces Research Institute of Medical Sciences (AFRIMS), Bangkok, Thailand; 6 Vaccine Trial Centre, Faculty of Tropical Medicine, Mahidol University, Bangkok, Thailand; 7 Department of Disease Control, C/O Ministry of Public Health, Nonthaburi, Thailand; 8 Department of Molecular Virology and Microbiology, Baylor College of Medicine, Houston, TX, United States of America; 9 Animal Models and Vaccine Section, National Cancer Institute, Bethesda, MD, United States of America; 10 Military HIV Research Program, Walter Reed Army Institute of Research, Silver Spring, MD, United States of America; 11 International Vaccine Institute, Gwanak-gu, Seoul, ROK; Vaccine Research Center, UNITED STATES

## Abstract

The concerns raised from adenovirus 5 (Ad5)-based HIV vaccine clinical trials, where excess HIV infections were observed in some vaccine recipients, have highlighted the importance of understanding host responses to vaccine vectors and the HIV susceptibility of vector-specific CD4 T cells in HIV vaccination. Our recent study reported that human Ad5-specific CD4 T cells induced by Ad5 vaccination (RV156A trial) are susceptible to HIV. Here we further investigated the HIV susceptibility of vector-specific CD4 T cells induced by ALVAC, a canarypox viral vector tested in the Thai trial RV144, as compared to Ad5 vector-specific CD4 T cells in the HVTN204 trial. We showed that while Ad5 vector-specific CD4 T cells were readily susceptible to HIV, ALVAC-specific CD4 T cells in RV144 PBMC were substantially less susceptible to both R5 and X4 HIV *in vitro*. The lower HIV susceptibility of ALVAC-specific CD4 T cells was associated with the reduced surface expression of HIV entry co-receptors CCR5 and CXCR4 on these cells. Phenotypic analyses identified that ALVAC-specific CD4 T cells displayed a strong Th1 phenotype, producing higher levels of IFN-γ and CCL4 (MIP-1β) but little IL-17. Of interest, ALVAC and Ad5 vectors induced distinct profiles of vector-specific CD8 vs. CD4 T-cell proliferative responses in PBMC, with ALVAC preferentially inducing CD8 T-cell proliferation, while Ad5 vector induced CD4 T-cell proliferation. Depletion of ALVAC-, but not Ad5-, induced CD8 T cells in PBMC led to a modest increase in HIV infection of vector-specific CD4 T cells, suggesting a role of ALVAC-specific CD8 T cells in protecting ALVAC-specific CD4 T cells from HIV. Taken together, our data provide strong evidence for distinct HIV susceptibility of CD4 T cells induced by different vaccine vectors and highlight the importance of better evaluating anti-vector responses in HIV vaccination.

## Author summary

Development of a safe and efficacious HIV vaccine is a critical global health priority. Recombinant viral vectors are an important platform for HIV vaccine delivery. Recent clinical trials testing candidate HIV vaccines based on Ad5 vectors failed and reported excess HIV infections in some vaccine recipients, underscoring the necessity to investigate HIV susceptibility of viral vector-specific CD4 T cells in HIV vaccination. By using PBMC samples from clinical trials that examined two important HIV vaccine vectors (canarypox viral vector ALVAC and human Ad5 vector), we here report that compared to Ad5 vector, the ALVAC-specific CD4 T cells are more resistant to HIV infection, providing evidence for distinct HIV susceptibility of CD4 T-cell populations induced by different HIV vaccine vectors. Our findings present new insights into our understanding of HIV vaccine-induced immunity and help improve the design and immune assessment of viral vectors for the development of HIV vaccines.

## Introduction

Over 30 years after the discovery of HIV as the causative agent of acquired immunodeficiency syndrome (AIDS), HIV/AIDS continues to be a significant challenge for global public health. More than 36 million people are currently living with HIV, with over 2 million new infections and 1 million AIDS-related deaths per year [1]. Development of a safe and effective HIV vaccine remains a high research priority. Recombinant viral vectors are an important platform for HIV vaccine development. To date, a number of HIV vaccine vectors derived from different viral families have been developed, including adenovirus [2] and poxvirus [3, 4]. Several clinical trials (Step and Phambili) testing candidate HIV vaccines based on human Ad5 vector (rAd5) have failed due to lack of efficacy and/or transiently increased HIV infections in some vaccinated individuals [5–7]. These unanticipated results from clinical trials have brought to light the importance of understanding host immune responses induced against viral vectors in HIV vaccination [8, 9].

CD4 T cells are central to host immunity by providing help signals to other components of the immune system [10]. The protective role of CD4 T cell responses has been documented for various pathogenic infections, including HIV [11–14]. However, CD4 T cells are also major target cells for HIV infection. During an antigen-specific immune response, activation and expansion of responding CD4 T cells is required [15], which is usually desired in most vaccine strategies but could become a potential problem in HIV vaccination due to the fact that HIV preferentially infects activated CD4 T cells [16–19]. Recent research from our group and others has shown that human CD4 T cells specific for different antigens differ in their susceptibility to HIV infection [20–26]. In particular, we have reported that human Ad5-specific CD4 T cells generated in response to both natural Ad5 infection and rAd5 vaccination are highly susceptible to HIV and are preferentially depleted in HIV-infected individuals [21]. Although potential mechanisms for Ad5 vector-associated excess HIV infections in the Step and Phambili studies are thought to be complex and could be affected by different factors such as the quantity, quality and *in vivo* localization of CD4 T cells induced during vaccination, our findings suggest that understanding the HIV susceptibility of vector-specific CD4 T-cell populations induced by different vaccine vectors may provide new insights into our understanding of host immunity in HIV vaccination.

In addition to rAd5, another important HIV vaccine vector that has been tested in late-stage clinical trials is ALVAC, a recombinant canarypox virus vector. The ALVAC prime/gp120 boost HIV vaccine regimen tested in the “Thai” RV144 trial demonstrated modest efficacy (~31%) [27]. Building upon the partial success of RV144, multiple ongoing trials further evaluating ALVAC-based HIV vaccine regimens are currently being conducted [28, 29]. In this study, we sought to understand anti-vector T cell responses with a focus on the phenotype and *in vitro* HIV susceptibility of vector-specific CD4 T cells induced by vaccination with ALVAC compared to Ad5. Cryopreserved peripheral blood mononuclear cells (PBMC) from RV144 vaccine recipients were analyzed in comparison with PBMC from HVTN204, a phase II trial evaluating rAd5-HIV vaccine (DNA prime/Ad5 boost) [30], using the *in vitro* HIV susceptibility assay reported in our previous studies [20, 21, 23]. We also measured vector-induced CD8 T-cell response in these PBMC samples. Our data show that vector-specific CD4 T cells induced by different HIV vaccine vectors manifest marked difference in their susceptibility to HIV infection; compared to Ad5-specific CD4 T cells in HVTN204 PBMC, the ALVAC-specific CD4 T cells in RV144 PBMC are substantially less susceptible to both R5 and X4 HIV infection *in vitro*. The differential HIV susceptibility between these two groups of vector-specific CD4 T cells is closely associated with their differences in phenotype, cytokine expression, and interestingly, the profiles of vector-specific CD8 vs. CD4 T-cell proliferative response induced by these two vectors.

## Results

### ALVAC-specific CD4 T cells are less susceptible to HIV infection *in vitro* than Ad5 vector-specific CD4 T cells

To compare the HIV susceptibility of different HIV vaccine vector-induced CD4 T cells (ALVAC vs. Ad5) in human vaccine recipients, we employed the *in vitro* HIV infection assay reported in our previous studies [20, 21, 23] (Summarized in S1 Fig**)**. In brief, PBMC samples of vaccine recipients in RV144 (ALVAC) and HVTN204 (Ad5 vector) were first stained with CFSE, a fluorescent dye used to track T-cell proliferation, and then stimulated with the corresponding empty vector for three days to induce the expansion of vector-reactive CD4 T cells, followed by infection with either CCR5-tropic (R5; US-1 strain) or CXCR4-tropic (X4; 92/UG/029 strain) HIV. Three days post-infection (dpi), flow cytometry was used to measure T-cell proliferation (indicated by decreased CFSE fluorescence intensity; CFSE-low) and HIV infectivity in vector-specific CD4 T cells (intracellular HIV p24^+^ rate in CFSE-low CD4 T cells) (S1A Fig). We have previously verified this *in vitro* system by demonstrating that the CFSE-low, proliferating CD4 T cells are mostly antigen specific (S1B Fig) and closely resemble their *in vivo* phenotypes (S1C Fig).

**Fig 1:**
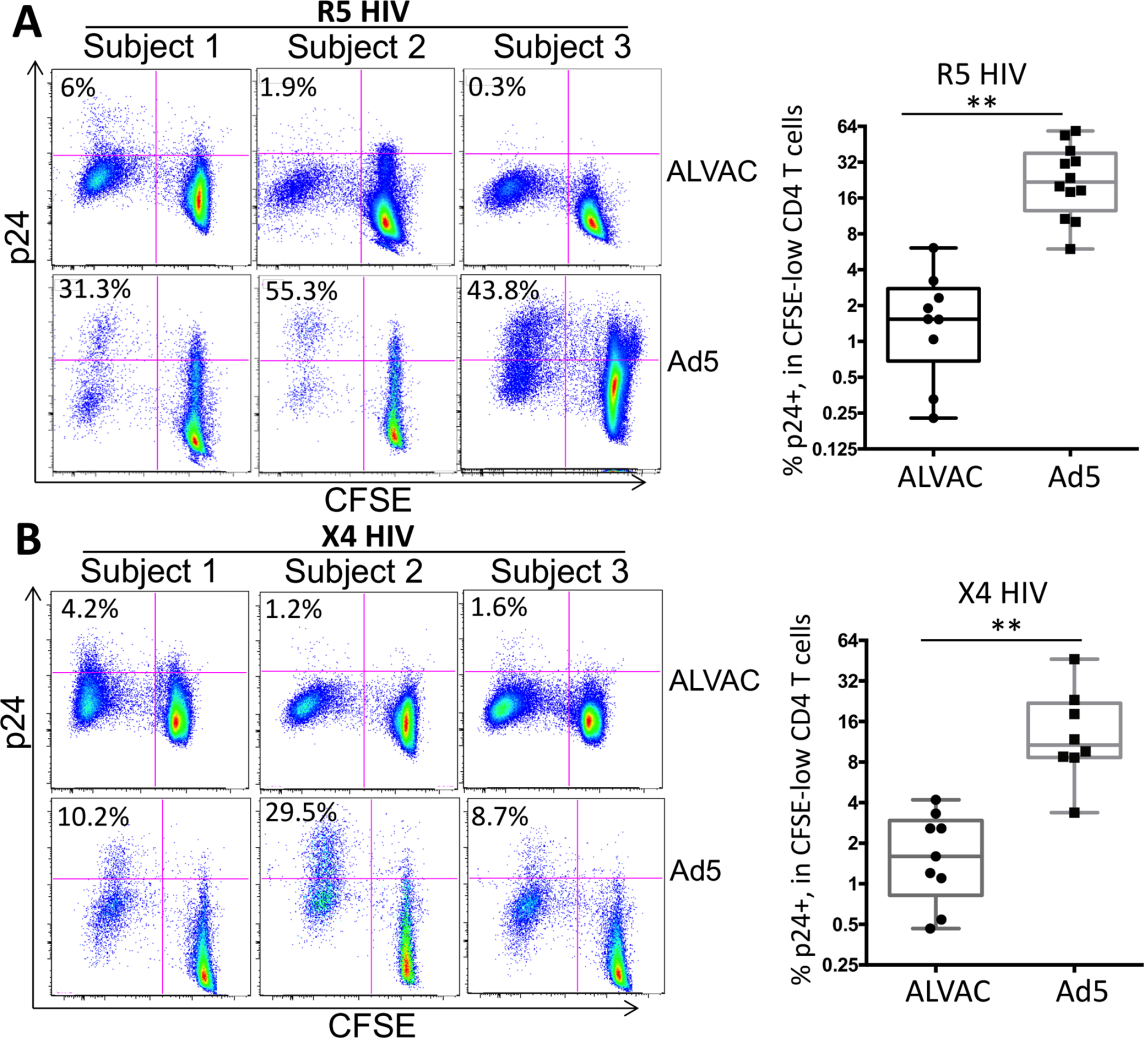
ALVAC-specific CD4 T cells are markedly less susceptible to HIV infection *in vitro* than Ad5 vector-specific CD4 T cells. PBMC collected from ALVAC- (RV144) or Ad5-vectored (HVTN204) HIV vaccine recipients were stained with CFSE and then re-stimulated with the recall vector antigen (ALVAC or Ad5) for three days before being infected with CCR5-tropic (US-1 strain) (**A**) or CXCR4-tropic (92/UG/029 strain) (**B**) HIV. HIV infection rate in vector-specific CD4 T cells was determined using flow cytometry to measure p24 expression 3 days post infection and expressed as the percentage of p24^+^ CFSE-low CD4 T cells. Representative flow cytometry plots shown at left are gated on CD3^+^CD8^-^ CD4 T cells. Statistical analysis was performed using an unpaired Student’s t test. *p ≤ 0.05, **p ≤ 0.01.

Based on this system, we first observed that both ALVAC and Ad5 vector induced significant levels of CD4 T-cell proliferation in PBMC of vaccine recipients (ALVAC for RV144 and Ad5 for HVTN204) (Fig 1). Regarding HIV susceptibility, we found that compared to Ad5 vector-induced CD4 T cells in HVTN204 PBMC, which were highly susceptible to R5 HIV infection (mean p24+%: 26.9%), the ALVAC-induced CD4 T cells in RV144 PBMC were markedly less susceptible to R5 HIV (mean p24+%: 1.27%) (p<0.01) (day 3 post-infection) (Fig 1A). We also monitored HIV infection in vector-induced CD4 T cells for up to 9 days post exposure and found that ALVAC-induced CD4 T cells remained resistant to HIV on day 9 post viral exposure (p24+: 0.5%), whereas Ad5 vector-specific CD4 T cells were still readily susceptible (p24+: 11.8%) (S2 Fig). Consistent with the results of R5 HIV infection, a similar lower susceptibility to X4 HIV (92/UG/029 strain) was also observed for ALVAC-induced CD4 T cells (mean p24+%: 1.82%) as compared to Ad5 vector-induced CD4 T cells (mean p24+%: 16.2%) (p<0.01) (Fig 1B). As controls, we showed that the two vectors induced very little T-cell proliferation in pre-vaccine PBMC of the same individuals (S3A Fig), suggesting that the T-cell proliferation observed in post-vaccine PBMC in our system were specific to vector with minimal non-specific proliferation. In addition, very little intracellular p24 (<0.1%) was detected in the same proliferating CD4 T cells when HIV was not added, supporting that intracellular p24 staining in our system is specific (S3B Fig). As another control, RV144 and HVTN204 PBMC were polyclonally activated by anti-CD3/CD28. We showed that anti-CD3/CD28-activated CD4 T cells in RV144 and HVTN204 PBMC were susceptible to HIV infection at comparable level (S4 Fig). Furthermore, we noted that in Ad5-stimulated PBMC, the CFSE-hi CD4 T cells appeared to be more sensitive to HIV as well compared to those in ALVAC-stimulated PBMC (Fig 1A). This might be related to the lower secretion of β-chemokines in the Ad5-stimulated PBMC culture, which will be presented later.

Transmitted founder virus (TFV) is important in HIV transmission. In addition to R5 US-1 and X4 92/UG/029 strains used, we also tested the susceptibility of vector-induced CD4 T cells to AD17 HIV molecular clone, a TFV [31, 32]. Consistently, we observed that ALVAC-induced CD4 T cells were also less susceptible to AD17 TFV infection (p24^+^ %: 0.6%) as compared to Ad5 vector-induced CD4 T cells (p24^+^ %: 3.8%) (S5 Fig), although the overall infectivity of AD17 TFV in these CD4 T cells was lower than that of the US-1 and 92/UG/029 strains (S5 Fig).

In vector HIV vaccination, insert-specific CD4 T cells are also induced in addition to vector-specific CD4 T cells. Therefore, we measured HIV susceptibility of vaccine Env-specific CD4 T cells using the same assay and found that unlike vector-specific CD4 T cells, Env-specific CD4 T cells in both RV144 and HVTN204 PBMC were readily susceptible to R5 and X4 HIV infection with no significant difference detected (S6 Fig). Taken together, these data suggest that the vector-specific CD4 T cells induced by different HIV vaccine vectors manifest marked differences in their susceptibility to both R5 and X4 HIV infection *in vitro*, with ALVAC-specific CD4 T cells being less susceptible than Ad5 vector-specific CD4 T cells.

### ALVAC-specific CD4 T cells express lower levels of HIV co-receptor CCR5 and CXCR4 than Ad5 vector-specific CD4 T cells

**Fig 2:**
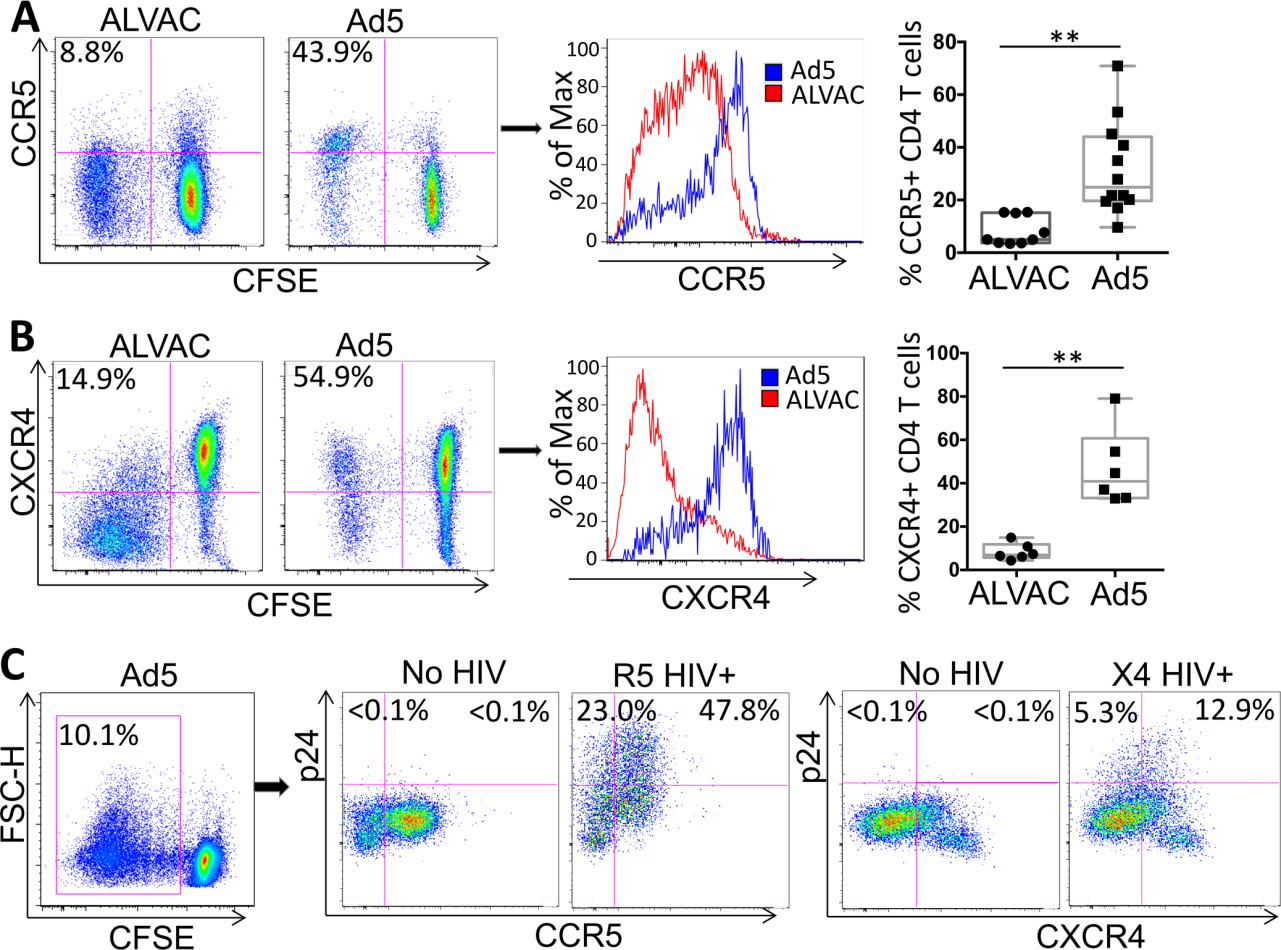
ALVAC vector-specific CD4 T cells express lower levels of the HIV co-receptors CCR5 and CXCR4 than Ad5 vector-specific CD4 T cells. PBMC of RV144 and HVTN204 vaccine recipients were stained with CFSE and stimulated with vector (ALVAC or Ad5) for 6 days. Surface expression of CCR5 (**A**) and CXCR4 (**B**) was measured by flow cytometry. Representative flow cytometry dot plots (left; gated on CD3+CD8- CD4 T cells) and histogram for co-receptor expression on ALVAC- and Ad5 vector-specific CD4 T cells are shown. Comparison of % CCR5+ or CXCR4+ vector-specific CD4 T cells from multiple subjects is shown (right). **(C)** HIV infection in co-receptor+ vs. co-receptor-, Ad5-specific CD4 T cells. CFSE-low, Ad5-specific CD4 T cells were gated for analysis (left). HIV infection rate (% p24+) in CCR5+ vs. CCR5- Ad5-specific CD4 T cells infected with R5 HIV (middle) or in CXCR4+ vs CXCR4- Ad5-specific CD4 T cells infected with X4 HIV (right) were shown. For both R5 and X4, no HIV infection was included as control to set p24 staining gate. Statistical analysis was performed using an unpaired Student’s t test; *p ≤ 0.05, **p ≤ 0.01.

We and others have shown that differential HIV susceptibility of human antigen-specific CD4 T cells can occur at both HIV entry and post-entry levels [20, 33]. An important factor that influences HIV infection of target cells at the entry level is the surface expression of the HIV co-receptors CCR5 and CXCR4. To understand potential mechanisms underlying the differential HIV susceptibility of ALVAC and Ad5 vector-specific CD4 T cells described above, we examined CCR5 and CXCR4 expression on these two groups of vector-specific CD4 T cells. We found that ALVAC-specific CD4 T cells expressed significantly lower frequencies of CCR5+ CD4 T cells (CCR5+%: 8.4 ± 1.8) than Ad5 vector-specific CD4 T cells (CCR5+%: 31.9 ± 5.1) (p<0.005) (Fig 2A). A similar difference was also observed for CXCR4 expression on ALVAC- and Ad5 vector-specific CD4 T cells (CXCR4+ % for ALVAC vs. Ad5: 8.3 ± 1.6 vs. 38.6 ± 7.4) (p<0.001) (Fig 2B). These data suggest that limited expression of CCR5 and CXCR4 represents an important mechanism for the lower susceptibility of ALVAC-specific CD4 T cells to R5 and X4 HIV, respectively, compared to Ad5 vector-specific CD4 T cells.

To better understand the relative contribution of co-receptor expression to the overall HIV susceptibility of vector-induced CD4 T cells in our system, we further analyzed HIV infection in co-receptor+ and co-receptor- (CCR5+/- and CXCR4+/-) subsets of Ad5-specific CD4 T cells as compared to that in ALVAC-specific CD4 T cells. Not surprisingly, we found that majority of HIV infection was observed in CCR5+ or CXCR4+ subsets of Ad5-specific CD4 T cells (Fig 2C). We also noted that the HIV infection rate in the CCR5- subset (p24+: 23%) or CXCR4- subset (p24+: 5.3%) of Ad5-specific CD4 T cells (Fig 2C) remained higher than the overall HIV infection rate in ALVAC-specific CD4 T cells (Fig 1). This data suggests that other factors may also contribute to the differential HIV susceptibility between Ad5- and ALVAC-specific CD4 T cells besides co-receptor expression.

### Innate antiviral state and immune activation status of ALVAC- and Ad5-specific CD4 T cells

**Fig 3:**
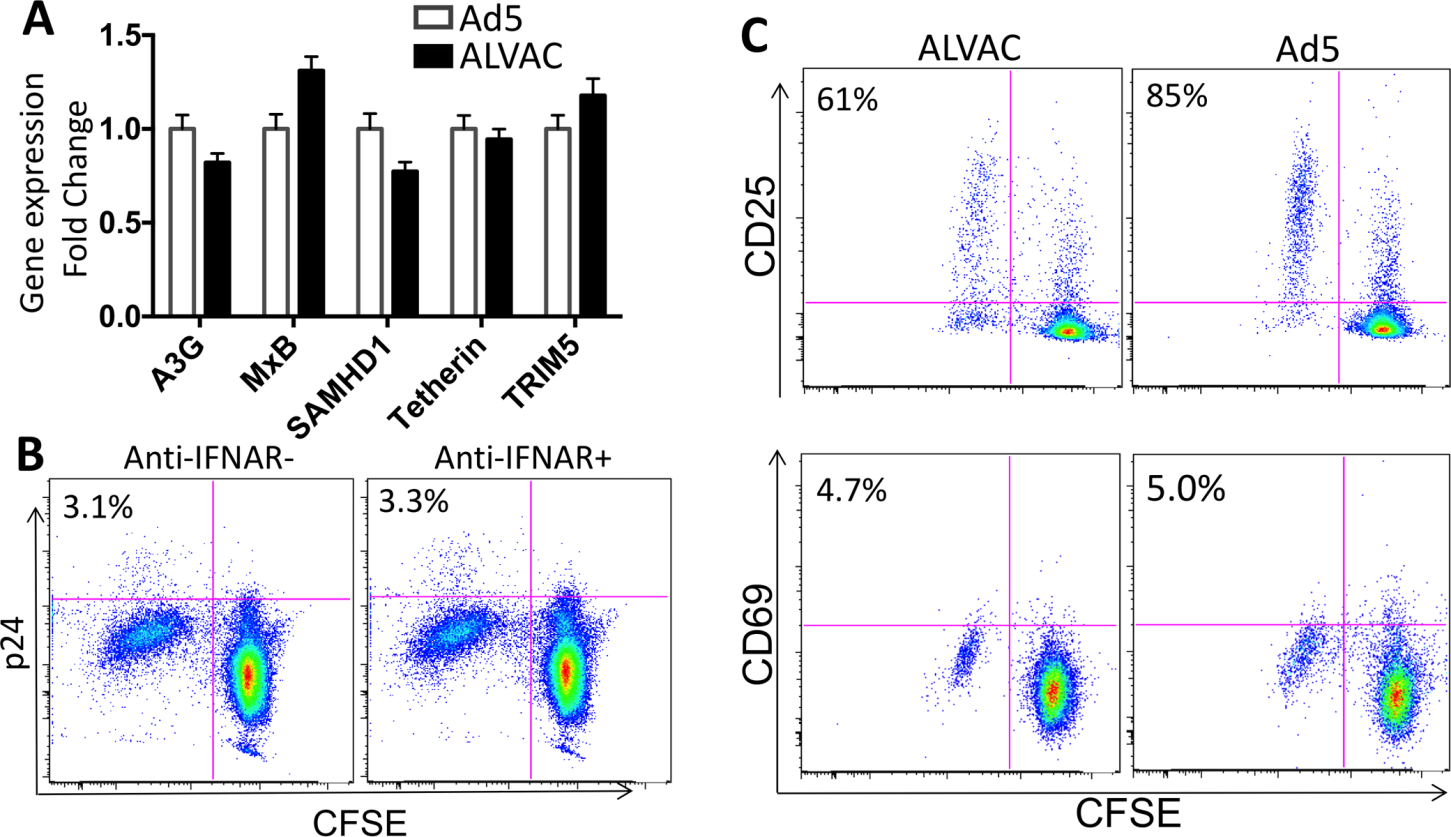
ALVAC- and Ad5-specific CD4 T cells show similar levels of innate antiviral gene expression and immune activation. **(A)** Relative expression of innate antiviral genes in ALVAC- and Ad5-specific CD4 T cells. RV144 and HVTN204 PBMC were CFSE-labeled and vector stimulated as described above. On day 6 ALVAC- and Ad5-specific CD4 T cells were sorted from PBMC based on CFSE-low and subjected to quantitative PCR for analysis of gene expression. The results were shown as fold change of ALVAC relative to Ad5. **(B)** HIV infection of ALVAC-specific CD4 T cells in RV144 PBMC in the presence or absence of anti-human IFNAR antibody blockade (gated on CD3+CD8- CD4 T cells). Number in each plot shows %p24+ in CFSE-low CD4 T cells. **(C)** Surface expression of T-cell activation markers CD25 (top) and CD69 (bottom) on ALVAC- vs. Ad5-specific CD4 T cells 6 days after stimulation with the corresponding vector (gated on CD3+CD8- CD4 T cells). Number in each plot shows % CD25+ or % CD69+ in CFSE-low CD4 T cells.

At the post-entry level of viral infection, HIV infectivity is associated with innate antiviral status and the activation state of target cells. Our recent study has demonstrated that ALVAC and Ad5 vector manifest distinct innate stimulatory properties with ALVAC being able to activate strong innate responses in antigen-presenting cells (APCs) [34]. This could potentially affect the antiviral status of CD4 T cells in vector-stimulated PBMC. We therefore compared the antiviral status of vector-specific CD4 T cells in our system. CFSE-low CD4 T cells were sorted from vector-stimulated PBMC and subjected to gene-expression analysis for antiviral genes and common HIV restriction factors, including A3G, MxB, SAMHD1, Tetherin and TRIM5. We found that expression of the genes was comparable between Ad5- and ALVAC-specific CD4 T cells (Fig 3A). Consistent with this result, blockade of type-I IFN signaling in ALVAC-stimulated PBMC [34] did not significantly alter the HIV infection in ALVAC-specific CD4 T cells (Fig 3B). These data suggest that the differential HIV susceptibility of vector-specific CD4 T cells may not be related to their innate antiviral status. Next, we assessed immune activation status of vector-specific CD4 T cells by examining the expression of T-cell activation markers (CD25 and CD69). While no significant difference CD69 expression was observed between ALVAC- and Ad5-specific CD4 T cells, Ad5-specific CD4 T cells appeared to express slightly higher level of CD25 than ALVAC-specific cells (Ad5 vs. ALVAC: 81% vs. 65%) (Fig 3C). This activation status of vector-specific CD4 T cells is generally consistent with their susceptibility to HIV infection.

### ALVAC-specific CD4 T cells display distinct phenotypic characteristics from Ad5 vector-specific CD4 T cells

**Fig 4:**
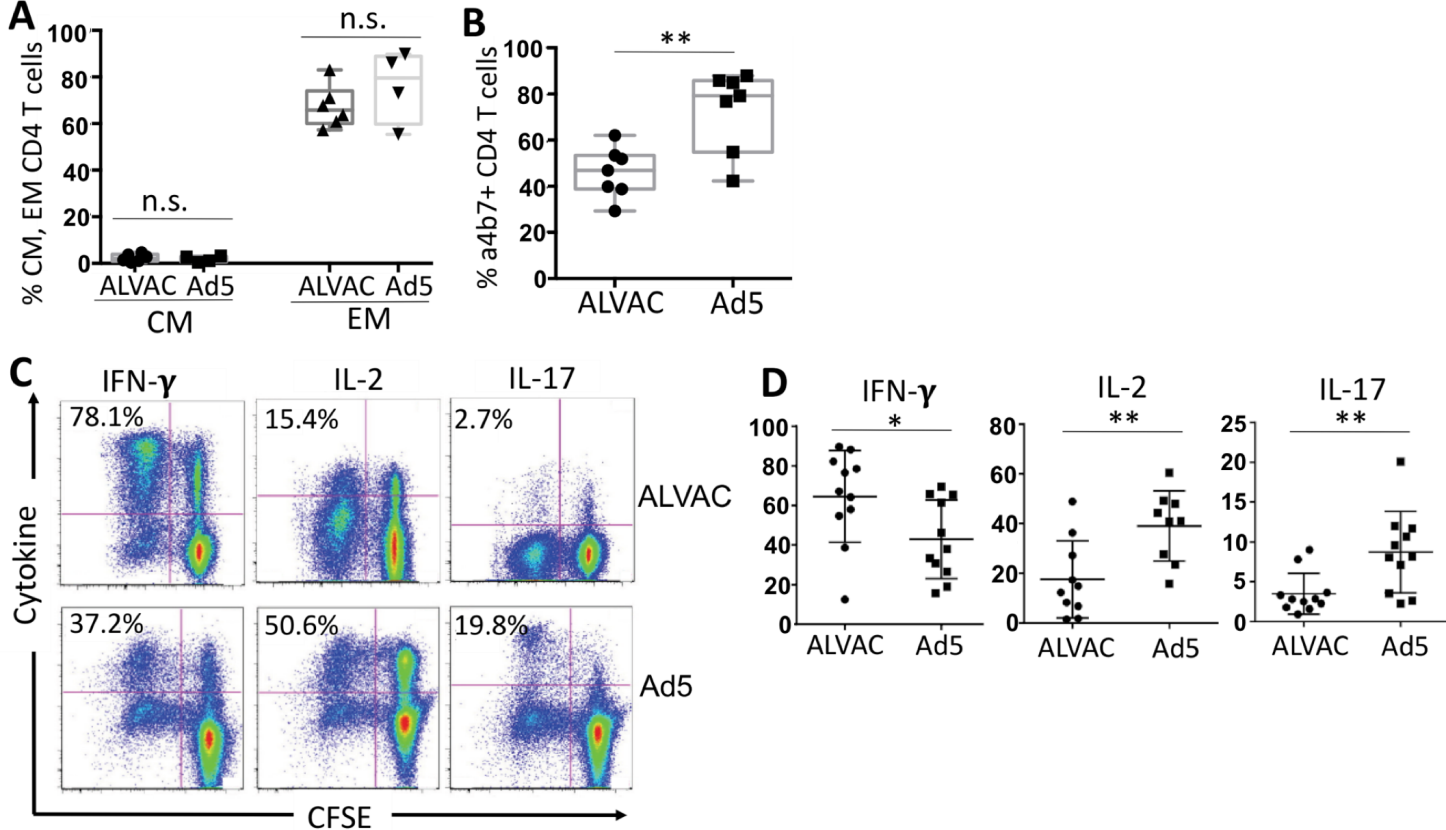
Phenotypic characterization of ALVAC- and Ad5 vector-specific CD4 T cells. PBMC from RV144 or HVTN204 vaccine recipients were stained with CFSE and re-stimulated with vector for 6 days. Phenotypes and cytokine profile of CFSE-low, vector-specific CD4 T cells were measured by flow cytometry. **(A)** Comparison for percent of central memory (CM: CCR7+CD45RO+) and effector memory (EM: CCR7-CD45R)+) subsets in CFSE-low, ALVAC- and Ad5 vector-specific CD4 T cells; **(B)** Comparison for α4β7+% in CFSE-low, ALVAC- and Ad5 vector-specific CD4 T cells; **(C)** Representative flow cytometric plots for cytokine expression (IFN-γ, IL-2, and IL-17) in CFSE-low, ALVAC-specific (top) or Ad5 vector-specific (bottom) CD4 T cells; **(D)** Comparison for cytokine expression in CFSE-low, vector-specific CD4 T cells (% cytokine+ CFSE-low) between ALVAC and Ad5 vector from multiple vaccine recipients (n = 11). n.s.: not significant, *p ≤ 0.05, **p ≤ 0.01.

Human antigen-specific CD4 T cell populations manifest different phenotypes in memory differentiation, T helper (Th) lineages, and cytokine profiles which are associated with their susceptibility to HIV infection [20–23, 25, 35, 36]. We next characterized major phenotypes of ALVAC- and Ad5 vector-specific CD4 T cells. Based on expression of CCR7 and CD45RO, human CD4 T cells can be categorized into central memory (CM: CCR7+CD45RO+) and effector memory subsets (EM: CCR7-CD45RO+). By focusing on the CFSE-low CD4 T cells, we found that both ALVAC- and Ad5 vector-specific CD4 T cells predominantly manifested an EM-like phenotype 2 weeks after the final vaccination, and no significant difference in memory phenotypes was observed between ALVAC- and Ad5 vector-specific CD4 T cells (Fig 4A). Mucosal homing is another important characteristic of CD4 T cells that influences HIV pathogenesis. Mucosal compartments represent a major site for HIV infection and CD4 depletion in HIV disease [37]. Integrin α4β7 is an important mucosal homing receptor, directing migration of CD4 T cells to gut mucosa [38]. We found that compared to Ad5 vector-specific CD4 T cells, which expressed high levels of α4β7 as reported in previous studies [16, 21], ALVAC-specific CD4 T cells expressed significantly lower levels of α4β7 (Fig 4B).

Next, we examined T-helper lineage and cytokine profile of ALVAC- and Ad5 vector -specific CD4 T cells. As described, CFSE-stained PBMC from vaccine recipients were stimulated with ALVAC or Ad5 vector to induce vector-specific CD4 T cell expansion. Since cytokine expression in activated T cells is usually transient and the CFSE-low, vector-specific CD4 T cells in our system undergo days of proliferation, in order to measure cytokine production in CFSE-low CD4 T cells the culture was re-stimulated with the global PMA/ionomycin stimulus on day 6 for cytokine *de novo* re-synthesis in T cells as we reported previously [21, 23]. Since Th17 CD4 T-cell subset has been shown to be highly susceptible to HIV as compared to Th1 subset [21, 23], we first measured expression of IFN-γ, IL-17 and IL-2 in vector-specific CD4 T cells (Fig 4C), and found that a significantly higher fraction of ALVAC-specific CD4 T cells expressed IFN-γ than Ad5 vector-specific CD4 T cells (64.6% ± 6.98 vs. 43.0% ± 5.96; p<0.05), typical of a strong Th1-like response. In contrast, a higher fraction of Ad5-specific CD4 T cells expressed IL-2 (39.0% ± 4.68 vs. 17.6% ± 4.90; p<0.01) and IL-17 (8.71% ± 1.55 vs. 3.50% ± 0.77; p<0.01), suggesting a mixed Th1/Th17 response (Fig 4C and 4D). This result is in agreement with our previous report that examined Ad5 vector-specific CD4 T cells in PBMC from the RV156A trial [21]. Therefore, since IL-17- and IL-2-producing CD4 T cells are known to be more susceptible to HIV infection than IFN-γ-producing CD4 T cells, this differential Th1 vs. Th1/Th17 phenotype for ALVAC- and Ad5-specific CD4 T cells is consistent with their susceptibility to HIV infection.

Besides Th1 and Th17 markers, we also examined other major T-cell associated phenotypes for vector-specific CD4 T cells, including T-follicular helper (Tfh), regulatory T cells (Treg) and PD-1 (T-cell exhaustion marker). First, we observed that a significant fraction of both ALVAC- and Ad5-specific CD4 T cells expressed IL-21, a lineage-specific cytokine for Tfh cells. However, unlike IFN-γ and IL-17, no significant difference in IL-21 expression was found between ALVAC- and Ad5-specific cells (S7A Fig). Further analysis identified that HIV infection in IL-21+ Tfh-like subset (p24+: 7.6%) was not higher than IL-21- subset (p24+: 10.3%) (S7B Fig), suggesting that in our system HIV does not preferentially infect Tfh-like CD4 subset [39]. Furthermore, we measured expression of Treg markers (CD25 and FoxP3) and exhaustion marker PD-1 in vector-specific CD4 T cells and found that, similar to Tfh, no significant difference in expression of Treg markers (S7C Fig) and PD-1 (S7D Fig) was observed between ALVAC- and Ad5-specific CD4 T cells. Altogether, these data suggest that Tfh, Treg and PD-1 phenotypes may not account for the differential HIV susceptibility of ALVAC- and Ad5-specific CD4 T cells in our system.

### Higher levels of MIP-1β in ALVAC-specific CD4 T cells contributes to their enhanced HIV resistance

**Fig 5:**
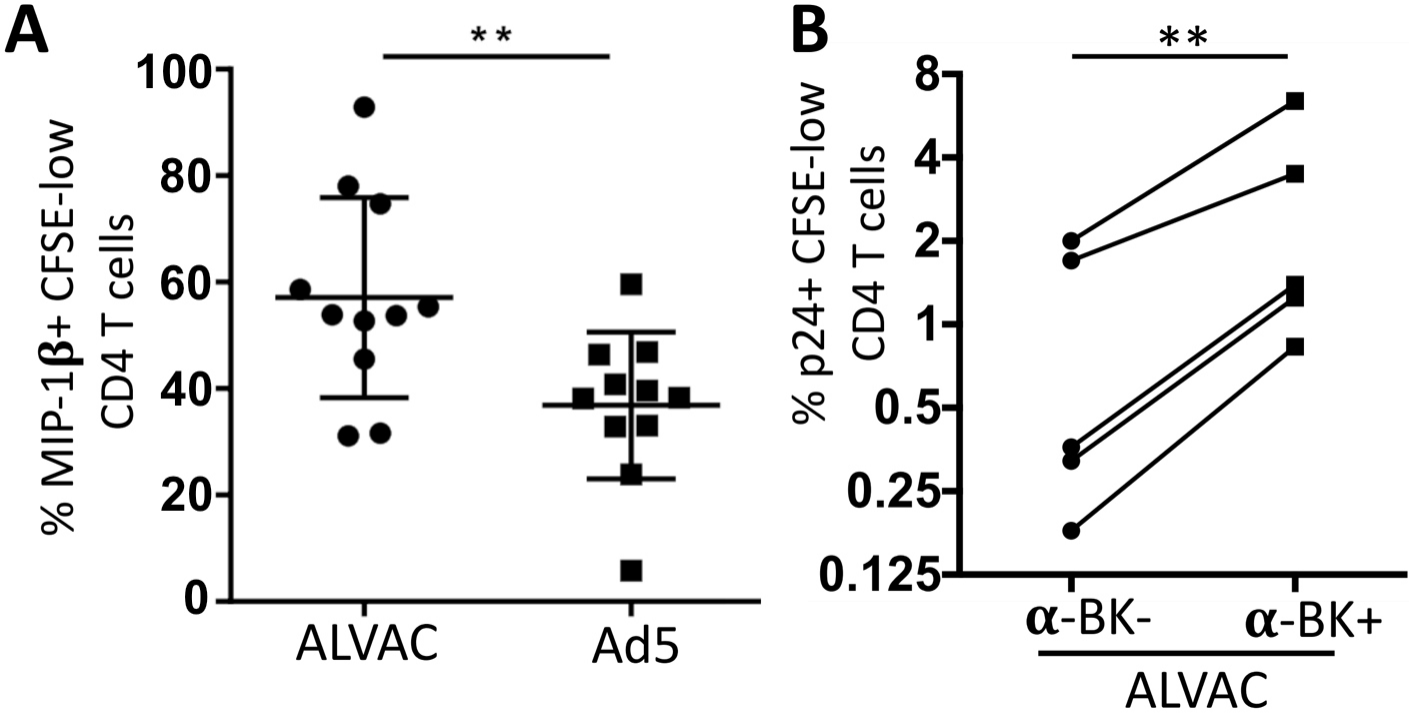
ALVAC-specific CD4 T cells produce higher levels of MIP-1β than Ad5-specific CD4 T cells, which contributes partly to their lower susceptibility to *in vitro* HIV infection. **(A)** MIP-1β expression in CFSE-low, vector-specific CD4 T cells was determined by intracellular cytokine staining and flow cytometric analysis as described above; results are expressed as % MIP-1β+ CFSE-low CD4 T cells (n = 11). **(B)** Impact of MIP-1β neutralization on HIV infection of ALVAC-specific CD4 T cells. PBMC were stained with CFSE and re-stimulated *in vitro* with ALVAC vector in the absence of presence of β-chemokine neutralizing antibodies (CCL3/4/5). 3 days after vector stimulation, PBMC were infected with R5 HIV, followed by measurement of HIV infection in vector-specific CD4 T cells (CFSE-low, CD4 T cells) on day 6 after initial vector stimulation. HIV infection was expressed as the percentage of p24+ in CFSE-low CD4 T cells (n = 4). *p ≤ 0.05, **p ≤ 0.01.

β-chemokines (MIP-1α, MIP-1β, and RANTES) are CCR5 ligands and can block CCR5-tropic (R5) HIV infection at entry level by competitively binding to CCR5 [33, 40, 41]. Therefore, we examined MIP-1β (CCL4) expression in the CFSE-low, vector-specific CD4 T cells. Not surprisingly, we found that a significantly higher fraction of ALVAC-specific CD4 T cells expressed MIP-1β than Ad5 vector-specific CD4 T cells (57.10% ± 5.67 vs. 36.84% ± 4.16; p<0.01) (Fig 5A). To evaluate the potential impact of β-chemokine production on HIV susceptibility of vector-specific CD4 T cells in our system, *in vitro* HIV infection (CCR5-tropic; US-1) was conducted in the presence of neutralizing antibodies against these β-chemokines (CCL3/MIP-1α, CCL4/MIP-1β, and CCL5/RANTES). We found that blocking β-chemokines could modestly, but significantly, increase the susceptibility of ALVAC-specific CD4 T cells to R5 HIV (p <0.01) (Fig 5B), suggesting a role for β-chemokines in protecting ALVAC-specific CD4 T cells from R5 HIV. However, we also found that even in the presence of β-chemokine neutralization, ALVAC-specific CD4 T cells were still significantly less susceptible to R5 HIV than Ad5 vector-specific CD4 T cells (Fig 5B; Fig 1A), suggesting that the higher production of β-chemokines contributes only partly to the lower susceptibility of ALVAC-specific CD4 T cells to HIV as compared to Ad5 vector-specific CD4 T cells in our system.

### ALVAC and Ad5 vectors elicit distinct profiles of vector-specific CD8 vs. CD4 T-cell proliferative response in PBMC of vaccine recipients

**Fig 6:**
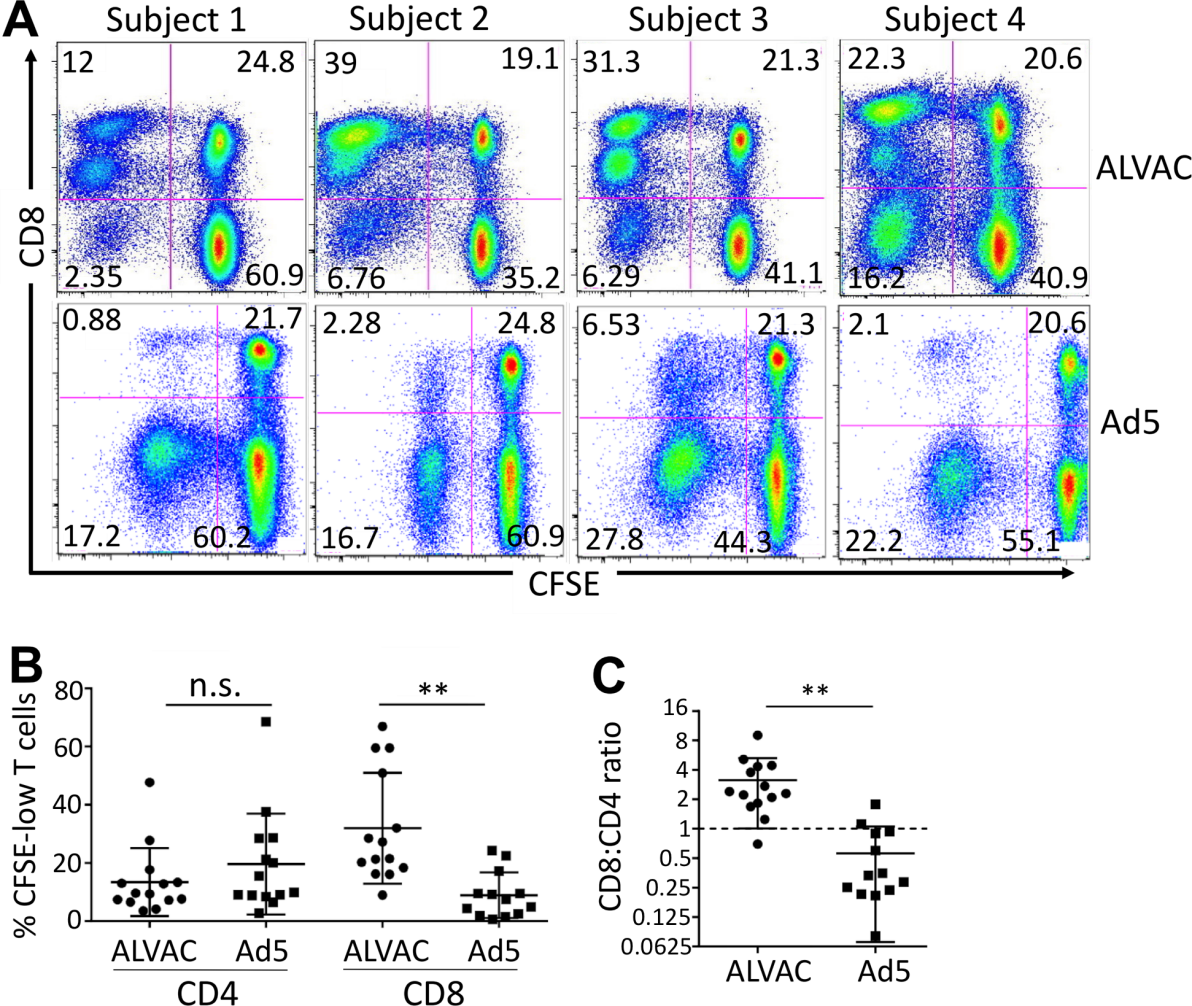
ALVAC elicits distinct profile of vector-specific CD8 vs. CD4 T-cell proliferative response compared to Ad5 vector. PBMC were stained with CFSE and re-stimulated with the corresponding vector for 6 days. **(A)** Representative flow cytometry plots for PBMC of multiple subjects showing vector-induced CD8 vs. CD4 T-cell proliferative responses in PBMC of RV144 (top) and HVTN204 (bottom) vaccine recipients. **(B)** Comparison for vector-specific CD8 and CD4 T-cell proliferative responses (% CFSE-low) in PBMC of RV144 and HVTN204 after corresponding vector stimulation. **(C)** Ratio of vector-specific CD8/CD4 T-cell proliferation in RV144 (ALVAC) and HVTN204 (Ad5) PBMC. Statistical analysis was performed using an unpaired Student’s t test; n = 14. *p ≤ 0.05, **p ≤ 0.01, ***p ≤ 0.001.

By simultaneous analyses of both CD8 and CD4 T cells, we found that ALVAC and Ad5 vector elicited distinct profiles of vector-specific CD8 vs. CD4 T-cell proliferative response in PBMC. ALVAC stimulated robust vector-specific CD8, but relatively weak vector-specific CD4, T-cell proliferation in RV144 PBMC, whereas Ad5 vector predominantly induced vector-specific CD4, but not CD8, T-cell proliferation in HVTN204 PBMC (Fig 6A). When we analyzed the cumulative results from multiple vaccine recipients (n = 14), although no significant difference in the magnitudes of vector-specific CD4 T-cell proliferation was observed between ALVAC and Ad5 (13.43 ± 3.118 vs 19.62 ± 4.633, respectively; p = 0.2776), ALVAC induced significantly higher levels of vector-specific CD8 T-cell proliferation in RV144 PBMC (31.94 ± 5.085 vs 8.908 ± 2.172; p = 0.0004) than Ad5 vector did in HVTN204 PBMC (Fig 6B). We further analyzed the ratio of vector-induced CD8 vs. CD4 T-cell proliferation within the same individuals and compared between ALVAC and Ad5 (Fig 6C), and found that ALVAC induced a much higher ratio of CD8/CD4 T-cell proliferation than Ad5 vector did (3.137 ± 0.5696 vs 0.5615 ± 0.1364; p = 0.0003) (Fig 6C). In contrast, the vaccine insert antigen envelope (Env) induced strong CD4 and weak CD8 T-cell proliferation in RV144 PBMC, but comparable levels of CD4 and CD8 T-cell proliferation in HVTN204 PBMC (S8 Fig), consistent with the results of Env-specific CD4/CD8 T-cell response measured by *ex vivo* ICS in previous studies [27, 30]. Taken together, these data suggest that ALVAC induces a distinct profile of vector-specific CD8 to CD4 T-cell proliferative response from that induced by Ad5 vector *in vitro*.

### ALVAC-, but not Ad5-, induced CD8 T cells inhibit the expansion of autologous vector-specific CD4 T cells

**Fig 7:**
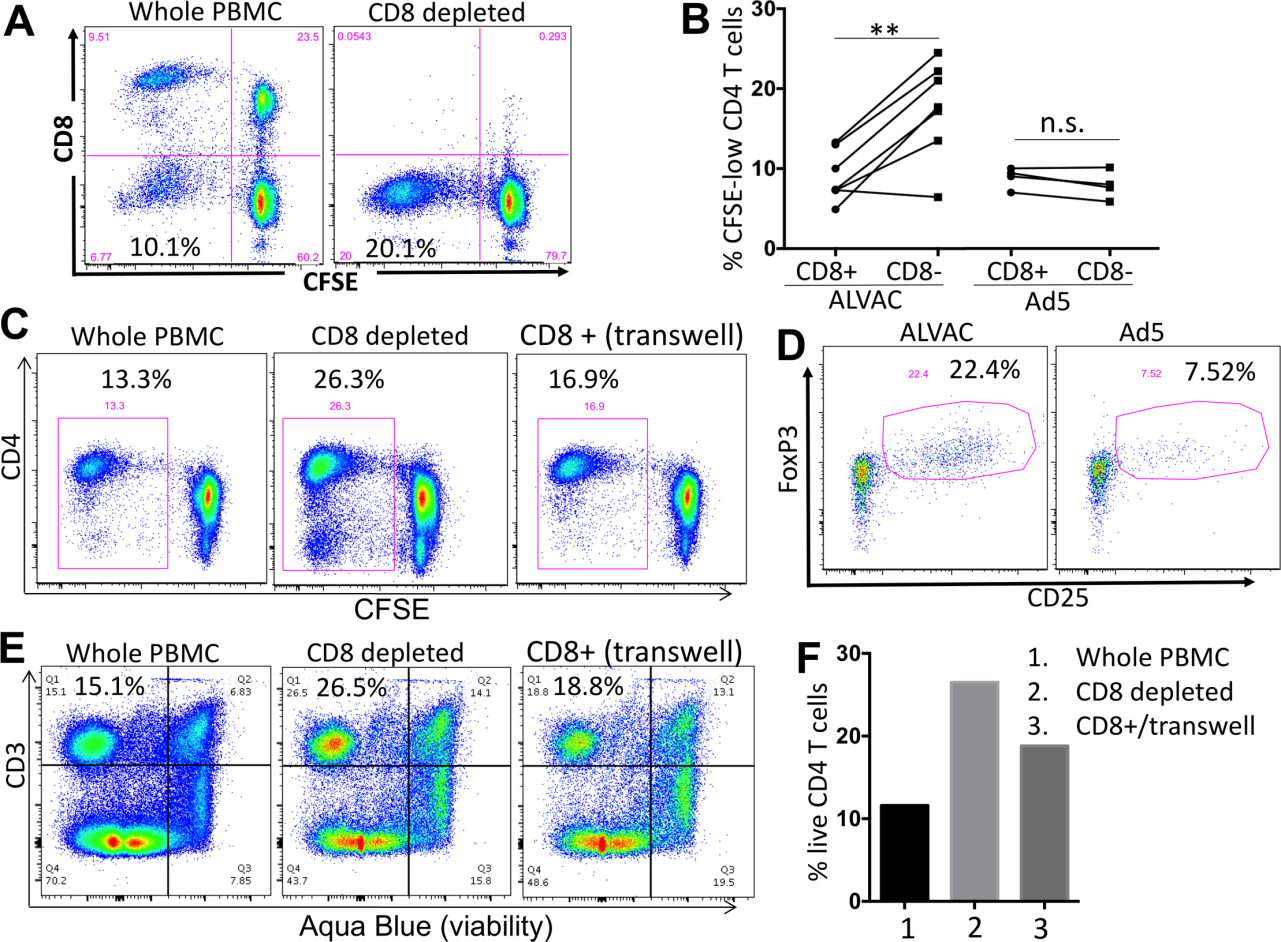
ALVAC-induced CD8 T cells inhibit the expansion of autologous vector-specific CD4 T cells. **(A)** CD8+ cells were depleted from PBMC of vaccine recipients using magnetic beads. CD8-depleted or whole PBMC were CFSE stained and re-stimulated with the appropriate vector for 6 days. Efficient CD8 depletion was verified by flow cytometry. Number in the bottom-left quadrant shows % CFSE-low, proliferating CD4 T cells in total CD4 T cells. **(B)** Comparison for vector-specific CD4 T cell proliferation (% CFSE-low) in PBMC with or without CD8 depletion (n = 7 for ALVAC; n = 4 for Ad5). **(C)** CD4 T-cell proliferation in RV144 PBMC 6 days after stimulation with ALVAC. Comparison of whole PBMC, CD8-depleted PBMC, and PBMC from which CD8 T cells were depleted and then added back to culture in trans-well (gated on CD3+ T cells). **(D)** CD25 and FoxP3 expression in ALVAC- versus Ad5-specific CD8 T cells 6 days after stimulation with the corresponding vector (gated on CD3+CD8+ CFSE-low T cells). **(E)** Flow cytometry plot and **(F)** bar graph showing CD4 T cell viability (% viable cells) in RV144 PBMC 3 days after stimulation with ALVAC (before significant T-cell proliferation occurs), as determined by Aqua Blue dye exclusion. Comparison of cell viability in whole PBMC, CD8-depleted PBMC, and PBMC from which CD8 T cells were depleted then added back to culture in trans-well. Statistical analysis was performed using an unpaired Student’s t test; n = 2 (Ad5) or 7 (ALVAC). n.s.: non-significant; *p ≤ 0.05, **p ≤ 0.01.

The importance of CD8 T cells in anti-HIV immunity, including control of viral replication and limiting HIV-infected CD4 T cells, has been well established [42–44]. In our system, we have observed low levels of CD4 T-cell proliferation in RV144 PBMC after ALVAC stimulation as compared to that in HVTN204 PBMC after Ad5 vector stimulation, which could possibly reflect the inhibition of CD4 T cell proliferation by ALVAC-induced CD8 T cells. Therefore, we next explored the potential impact of vector-induced CD8 T cells on vector-specific CD4 T cell proliferation. CD8 T cells were depleted from PBMC using magnetic cell sorting (MACS) prior to CFSE staining and vector re-stimulation. Efficient depletion of CD8 T cells from PBMC was confirmed (Fig 7A). Subsequently, proliferation of CD4 and CD8 T cells in the whole or CD8-depleted PBMC was measured on day 6 by flow cytometry. We showed that depletion of CD8 T cells from ALVAC-stimulated PBMC led to a significant increase in the proliferation of ALVAC-specific CD4 T cells in RV144 PBMC (p = 0.0068), whereas no such effect was seen in CD8-depleted HVTN204 PBMC when stimulated by Ad5 vector (p = 0.1747) (Fig 7B). These results suggest that, unlike Ad5 vector-induced CD8 T cells, ALVAC-induced CD8 T cells can inhibit the expansion of autologous vector-specific CD4 T cells in PBMC.

To explore potential mechanisms by which ALVAC-stimulated CD8 T cells inhibit autologous ALVAC-specific CD4 T-cell proliferation, we conducted trans-well experiments where CD8 T cells were first depleted from PBMC and then added back to the culture in trans-well. We found that addition of CD8 T cells in trans-well could largely, though not completely, restore the inhibitory effect of CD8 T cells on ALVAC-specific CD4 T-cell proliferation (from 26.3% to 16.9%, compared to 13.3% for whole PBMC) (Fig 7C), suggesting that CD8 T cells inhibit ALVAC-specific CD4 proliferation via a cell-contact-independent mechanism. CD25+FoxP3+ regulatory CD8 T cells are an emerging CD8 subset with strong suppressive activities [45]. We measured CD25 and FoxP3 expression in vector-activated CD8 T cells on day 6 after initial vector stimulation and found that a much higher fraction of ALVAC-activated CD8 T cells were CD25+FoxP3+ (22.4%) as compared to Ad5-activated CD8 T cells (7.52%) (Fig 7D), suggesting that ALVAC-induced CD25+FoxP3+ CD8 T cells could play a role in inhibition of autologous vector-specific CD4 T-cell proliferation.

In addition to CD4 T cell inhibition, we also explored potential cytolytic effects of CD8 T cells from RV144 vaccine recipients on autologous CD4 T cells in response to ALVAC stimulation. Three conditions of RV144 PBMC were prepared as described above, including whole PBMC, CD8-depleted PBMC, and CD8 T cell addition back to trans-well culture (Fig 7E). On day 3 after ALVAC stimulation, before significant cell proliferation occurred in the culture (S9 Fig), the viability of total cells (CD3+ T cells and CD3- non-T cells) was measured by flow cytometry based on aqua blue staining (Fig 7E). We observed that compared to the whole PBMC that had only 15.1% live T cells, CD8-depleted PBMC had higher levels of live T cells (26.5%) (Fig 7E and 7F). Addition of the depleted CD8 T cells back to the trans-well culture decreased the level of live T cells (18.8%) (Fig 7E and 7F). The percent of live CD4 T cells (after subtracting CD8 T cells from the total live CD3+ T cells) in each condition was summarized and shown in Fig 7F. This data suggests that in ALVAC-stimulated PBMC, CD8 T cells can manifest a cytotoxic effect on the autologous CD4 T cells, which involves a cell-contact-independent mechanism. This cytotoxic effect of CD8 T cells may also contribute to the overall inhibition of ALVAC-specific CD4 T-cell expansion in our system.

### ALVAC-, but not Ad5-, induced CD8 T cells limit HIV infection of autologous vector-specific CD4 T cells

**Fig 8:**
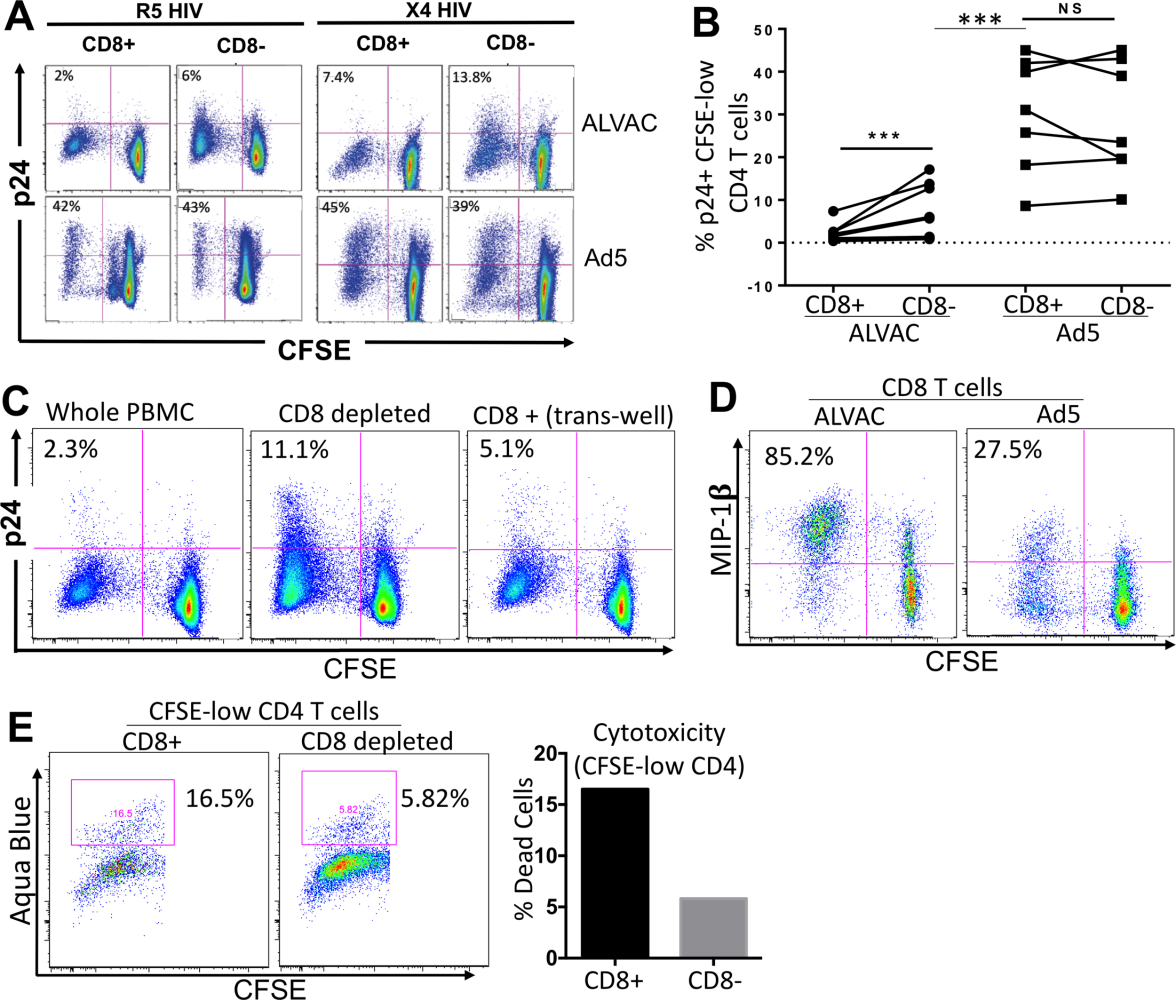
CD8 depletion increases HIV susceptibility of ALVAC-specific CD4 T cells. **(A)** Representative flow cytometry plots showing HIV infection in CFSE-low, vector-specific CD4 T cells in whole (CD8+) or CD8-depleted (CD8-) PBMC. Whole and CD8-depleted PBMC were CFSE stained and stimulated with vector antigen for 3 days before being infected with CCR5- or CXCR4-tropic HIV. HIV infection rate was determined using flow cytometry to measure intracellular p24 and expressed as the percentage of p24+ in CFSE-low CD4 T cells. CD3+CD8- T cells were gated for analysis. **(B)** Comparison for HIV infection rates in CFSE-low vector-specific CD4 T cells (% p24+) in whole or CD8-depleted PBMC from multiple vaccine recipients. **(C)** HIV infection (% p24+) in ALVAC-specific CD4 T cells in whole PBMC, CD8-depleted PBMC or PBMC from which CD8 T cells have been depleted and then added back to culture in trans-well (gated on CD3+CD8- CD4 T cells). % p24+ in CFSE-low cells was shown. **(D)** MIP-1β expression in ALVAC- versus Ad5-specific CD8 T cells 6 days after stimulation with the corresponding vector (gated on CD3+CD8+ T cells). **(E)** Flow cytometry plot (left) and bar graph (right) showing the viability of ALVAC-specific CD4 T cells (based on Aqua Blue staining) 6 days after vector stimulation with or without CD8 T-cell depletion (gated on CD3+CD8- CD4 T cells). Statistical analysis was performed using an unpaired Student’s t test; *p ≤ 0.05, **p ≤ 0.01, ***p ≤ 0.001.

In the context of vector HIV vaccination, it has been speculated that vector-induced CD4 T cells can be potential targets for HIV, which may affect the risk of HIV acquisition in vaccine recipients and overall outcome of vaccination [8]. Therefore, limiting the numbers and/or HIV susceptibility of vector-induced CD4 T cells in HIV vaccination is thought to be critical. We next explored the impact of vector-induced CD8 T cells on HIV susceptibility of autologous vector-specific CD4 T cells in PBMC, by using the above CD8-depletion assay. Whole or CD8-depleted PBMC were CFSE-labeled, and stimulated with vector antigen for 3 days, followed by infection with R5 or X4 HIV. Three days after infection, HIV infectivity in vector-specific CD4 T cells was measured by flow cytometry based on intracellular HIV p24 expression in CFSE-low CD4 T cells. We found that compared to whole PBMC, depletion of CD8 T cells from ALVAC-stimulated PBMC led to considerable increase in both R5 and X4 HIV infection of ALVAC-specific CD4 T cells (R5 HIV for CD8+ and CD8-: 2% vs. 6%; X4 HIV for CD8+ and CD8-: 7.4% vs. 13.8%) (Fig 8A). Analyses of PBMC from multiple subjects showed strong statistical significance between whole and CD8-depleted PBMC (p = 0.0006) (Fig 8B); in contrast, depletion of CD8 T cells in Ad5 vector-stimulated PBMC (HVTN204) had no significant impact on HIV infection rate of Ad5 vector-specific CD4 T cells (Fig 8A and 8B). Of interest, it should be noted that even in the absence of CD8 T cells (CD8 depletion), ALVAC-specific CD4 T cells were still significantly less susceptible to HIV infection than Ad5 vector-specific CD4 T cells (5.63 ± 1.84 vs 28.56 ± 5.16; p = 0.0002) (Fig 8B), suggesting that CD8 T cells contributed only partly to the low HIV susceptibility of ALVAC-specific CD4 T cells as compared to Ad5 vector-specific CD4 T cells. These data indicate that unlike Ad5 vector, ALVAC may induce vector-specific CD8 T cells that can not only inhibit the expansion of autologous vector-specific CD4 T cells, but also limit their susceptibility to HIV infection.

CD8 T cells can control viral infections through various mechanisms, including cytolytic activity and the secretion of soluble HIV-suppressive factors [46]. We next characterized potential mechanisms underlying CD8-mediated HIV inhibition in autologous ALVAC-specific CD4 T cells. First, we observed that CD8 depletion did not significantly affect the expression of CCR5 and T-cell activation markers (CD25 and CD69) on ALVAC-specific CD4 T cells (S10 Fig). We then performed a similar CD8 trans-well experiment to explore if the HIV inhibition by CD8 T cells is dependent of cell contact or soluble factors. We found that CD8 T cells could still inhibit R5 HIV infection in ALVAC-specific CD4 T cells even in the absence of direct cell contact (p24% for CD8- vs. trans-well CD8+: 11.1% vs. 5.1%) (Fig 8C), indicating that soluble HIV suppressive factors may play a role in this process. Consistent with this observation, we found that compared to Ad5 vector, ALVAC-induced CD8 T cells produced markedly higher levels of MIP-1β (MIP-1β+ % in Ad5 vs. ALVAC-induced CD8 T cells: 27.5% vs. 85.2%) (Fig 8D). Since in ALVAC-stimulated PBMC, high levels of CD8 T cells were induced (Fig 6), and β-chemokines were shown to mediate R5 HIV inhibition in ALVAC-specific CD4 T cells in our system (Fig 5), secretion of more β-chemokines might represent a mechanism for HIV inhibition in ALVAC-specific CD4 T cells by CD8 T cells. Lastly, we observed that compared to CD8-depleted PBMC, the presence of CD8 T cells in PBMC led to higher level of cell death (based on aqua blue staining) in CFSE-low, ALVAC-specific CD4 T cells (aqua blue staining in CD8+ vs. CD8-: 16.5% vs. 5.82%) (Fig 8E). This data suggests that the cytotoxic effects of CD8 T cells may also contribute to overall HIV inhibition in ALVAC-specific CD4 T cells.

### ALVAC-induced CD8 T cells manifest a stronger antiviral and cytotoxic phenotype than Ad5 vector-induced CD8 T cells

**Fig 9:**
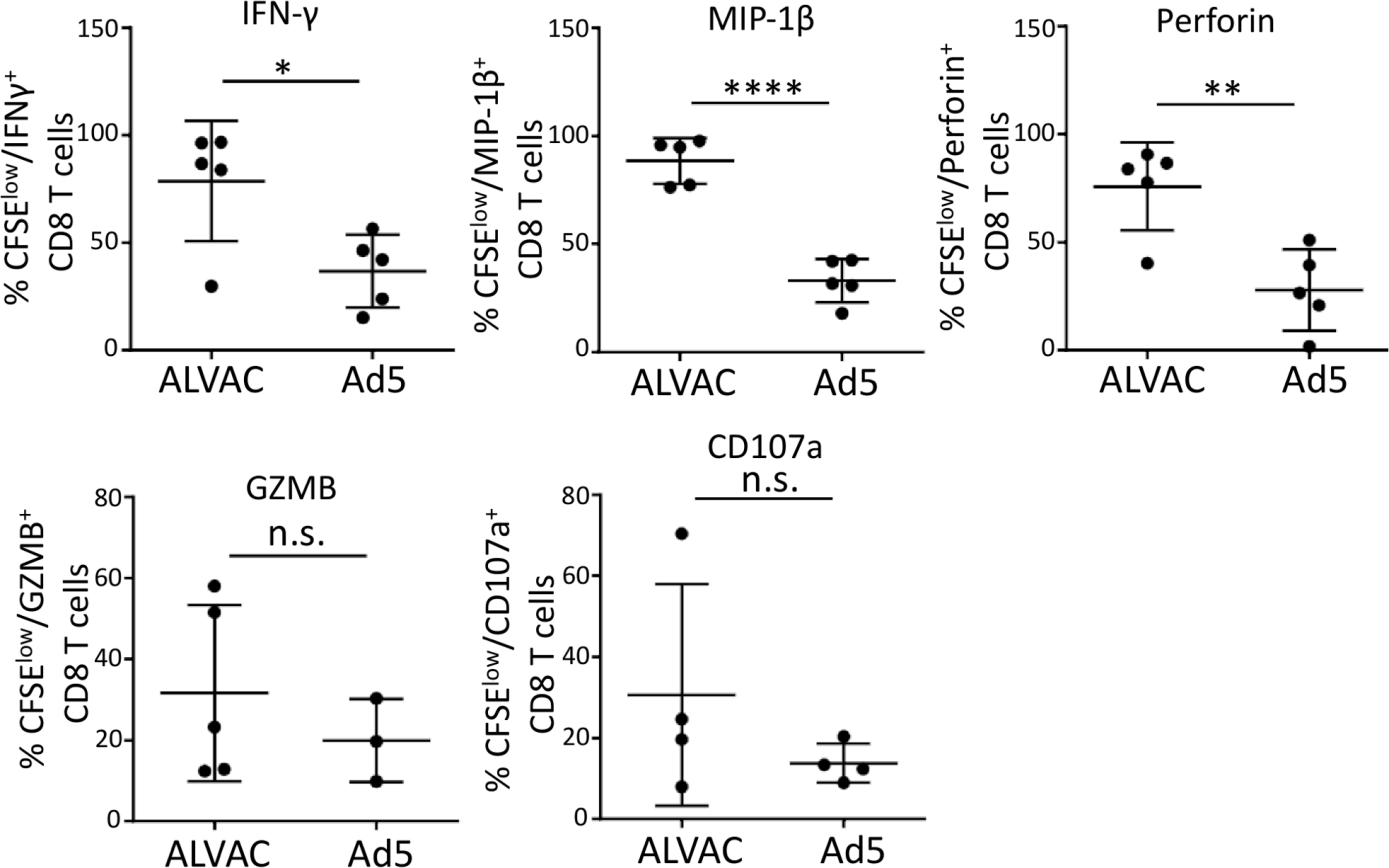
ALVAC-induced CD8 T cells manifest stronger antiviral and cytotoxic phenotype than Ad5 vector-induced CD8 T cells. PBMC of vaccine recipients were stained with CFSE and then stimulated with vector antigen for 6 days, followed by brief PMA/Ionomycin re-stimulation (6 hours) for cytokine/effector molecule re-synthesis. Intracellular staining and flow cytometry were used to measure the production of IFN-γ, MIP-1β, perforin, granzyme B (GZMB), and CD107a; results are expressed as % cytokine+ in CFSE-low CD8 T cells. Statistical analysis was performed using an unpaired Student’s t test; n = 3–5. n.s.: not significant; *p ≤ 0.05, **p ≤ 0.01, ***p ≤ 0.001, ****p ≤ 0.0001.

Lastly, we characterized the poly-functional profile of ALVAC- and Ad5 vector-induced CD8 T cells by examining expression of antiviral and cytolytic effectors. CFSE-stained PBMC from RV144 or HVTN204 were re-stimulated with ALVAC or Ad5 vector, respectively, as described above. Six days after stimulation, cells were briefly treated with PMA and ionomycin for 6 hours to induce *de novo* re-synthesis of cytokines or effector molecules. Expression of IFN-γ, MIP-1β, CD107a, granzyme B (GZMB), and perforin in CFSE-low, vector-induced CD8 T cells was measured by flow cytometry. We found that compared to Ad5 vector, significantly higher percentages of ALVAC-induced CD8 T cells expressed IFN-γ (78.74 ± 12.50 vs 36.86 ± 7.57; p = 0.0210), MIP-1β (88.38 ± 4.753 vs 33.00 ± 4.51; p < 0.0001) and perforin (75.86 ± 9.139 vs 27.91 ± 8.369; p = 0.0047) (Fig 9). No significant difference in expression of GZMB (ALVAC vs. Ad5: 31.60 ± 9.720 vs 19.94 ± 5.913; p = 0.4261) and CD107a (ALVAC vs. Ad5: 15.32 ± 6.853 vs 6.893 ± 1.199; p = 0.2713) was observed between ALVAC- and Ad5-induced CD8 T cells (Fig 9). Altogether, these data suggest that ALVAC-induced CD8 T cells manifest a stronger antiviral and cytolytic phenotype than Ad5 vector-induced CD8 T cells.

## Discussion

In the present study, by using PBMC samples from two important HIV vaccine trials, we investigated host anti-vector T-cell responses induced by ALVAC and Ad5 vector in human vaccine recipients with a focus on the HIV susceptibility of vector-specific CD4 T cells. Our major finding is that different HIV vaccine vector-induced CD4 T cells manifest distinct susceptibility to HIV infection; while Ad5 vector-specific CD4 T cells are readily susceptible to HIV [21], ALVAC-specific CD4 T cells in RV144 PBMC are more resistant to both R5 and X4 HIV infection. Associated with this are the differences in phenotypes and cytokine profiles of these two groups of vector-specific CD4 T cells. Another major finding of our study is that in contrast to the lack of vaccine insert-specific CD8 T-cell response reported from the RV144 trial [27, 47], we demonstrate that ALVAC vector induces strong proliferative response of vector-specific CD8 T cells, which can limit the proliferation and HIV susceptibility of the autologous ALVAC-specific CD4 T cells.

The unexpected outcomes of human trials testing HIV vaccine regimens involving different viral vectors have suggested that assessment of both protective and potentially detrimental immune responses induced by vaccination is important [8, 48]. Development of a safe and efficacious HIV vaccine poses a unique challenge in that HIV infects the very CD4 T cells which are usually required to mount an effective adaptive response; this is of especial concern for viral vector vaccines because the expansion of vector-specific CD4 T cells following immunization can provide potential HIV target cells [8, 21], while presumably not contributing to anti-HIV immunity. From this point of view, it would be advantageous to employ vectors which generate fewer and/or less HIV-susceptible vector-specific CD4 T cells. Human CD4 T cells specific for different antigens or pathogens manifest differential susceptibility to HIV [20–26]. Our previous study has reported that human Ad5-specific CD4 T cells induced by natural infection or rAd5 vaccination are more susceptible to HIV infection [21]. This finding suggests that although Ad5 vectors have been commonly employed for vaccine development due to their potent immunogenicity [49], the advantages of Ad5 as a vector may be dampened by the high HIV susceptibility of CD4 T cells it induces. Our current study shows that unlike Ad5 vector, the vector-specific CD4 T cells induced by ALVAC in RV144 are markedly less susceptible to HIV infection (Fig 1). This finding is relevant to HIV vaccine development, considering that in the context of HIV vaccination, if vaccine-induced protective immunity is comparable between different vaccine regimens, the relative HIV susceptibility of vector-specific CD4 T cells may be an important factor that can affect the overall outcome of HIV vaccination. Future studies are being planned to examine the HIV susceptibility of CD4 T cells induced by other important HIV vaccine vectors, especially the adenovirus rare serotypes Ad26 and Ad35.

Parameters that influence HIV acquisition risk in HIV vaccination are thought to be complex, among which the level, quality (e.g. phenotypes, cytokine profile, and HIV susceptibility) and *in vivo* localization of induced CD4 T cells play important roles. Our data suggest that the high HIV susceptibility of Ad5 vector-specific CD4 T cells may be a contributing factor for the observed excess HIV infections in some Ad5-HIV vaccine recipients [5–7]. In addition, our ongoing studies examining *in vivo* localization and phenotypes of CD4 T cells following ALVAC and Ad5 immunization show that ALVAC immunization induces substantial lower levels of CCR5+CD4+ and CCR5+ α4β7+CD4+ T cells in various immune compartments, especially in the gut mucosa, of the immunized mice as compared to Ad5 immunization. Based on these findings, we propose that to better understand immune parameters associated with HIV acquisition risk in vector HIV vaccination, future studies are warranted to more thoroughly assess the frequency, quality and *in vivo* localization of vaccine-induced CD4 T cells in animal models and/or human trials.

HIV infection of antigen-specific CD4 T cells can be regulated at both entry and post-entry levels, and is closely associated with the phenotypic and functional characteristics of these CD4 T cells [20, 25, 33, 35]. CCR5 and CXCR4 as HIV entry co-receptors play major roles in regulating the susceptibility of target cells to HIV at entry level [50]. Our data show that ALVAC-specific CD4 T cells express markedly lower levels of CCR5 and CXCR4 than Ad5 vector-specific CD4 T cells (Fig 2), providing an explanation for the lower HIV susceptibility of ALVAC-specific CD4 T cells. We further identified that HIV infection rate in CCR5-/CXCR4- subset of Ad5-specific CD4 T cells remained higher than that in ALVAC-specific CD4 T cells (Fig 2C), suggesting that factors other than co-receptor expression are also involved in regulating the differential HIV susceptibility of vector-specific CD4 T cells in our system. Regulation of HIV co-receptor expression on target cells has been investigated previously in HIV pathogenesis [33, 51]. However, currently little is known about co-receptor regulation in HIV vaccination. Evidence from our ongoing studies suggests that innate signals derived from vector-infected APCs play a role in regulating CCR5 on CD4 T cells. Further understanding mechanisms that regulate HIV co-receptor expression on vaccine-induced cells is an interesting topic and should be pursued in future studies.

Another important factor that regulates HIV infection of CD4 T cells at entry level is β-chemokines, including CCL3 (MIP-1α), CCL4 (MIP-1β), and CCL5 (RANTES) [33, 40, 41]. Our data show that compared to Ad5 vector, ALVAC-induced T cells (CD4 and CD8) produce much higher levels of β-chemokines (MIP-1β) (Fig 5A); however, interestingly, neutralization of β-chemokines in ALVAC-stimulated PBMC only slightly increased HIV infection in ALVAC-specific CD4 T cells (Fig 5B), suggesting a modest role of β-chemokines in this process. In addition to co-receptors and β-chemokines, cytokine profiles of CD4 T cells are closely associated with HIV infection. It has been shown that IL-17-producing CD4 T cells are more susceptible to HIV than IFN-γ-producing CD4 T cells [20, 21, 23, 52, 53]. In our study, we demonstrate that while Ad5 vector-specific CD4 T cells manifest a mixed Th1/Th17 phenotype, producing high levels of IL-17 and IFN-γ [21], ALVAC-specific CD4 T cells display a polarized Th1-like phenotype, producing high level of IFN-γ but very little IL-17 (Fig 4C and 4D). This different cytokine profile of ALVAC- and Ad5-specific CD4 T cells is consistent with their susceptibility to HIV infection in our system.

CD8 T cells play important roles in anti-HIV immunity, including control of HIV replication and limiting HIV-infected cells [43, 44]. An interesting observation in the current study is that ALVAC and Ad5 vector stimulate distinct CD8 vs. CD4 T-cell proliferative responses; Ad5 vector stimulate predominantly CD4 T-cell proliferation, whereas ALVAC stimulate strong CD8 T-cell proliferation (Fig 6). This finding is somewhat unexpected since the ALVAC/gp120 vaccine regimen in the RV144 trial was reported to elicit a weak insert-specific CD8 response [27], whereas Ad5-HIV vaccines have been shown to induce a strong insert-specific CD8 response [5, 6, 30]. These findings suggest that the induction of anti-vector and anti-insert T-cell responses in vector HIV vaccination may be differentially regulated. In this study, mechanisms for differential stimulation of vector-specific CD8 vs. CD4 T-cell proliferation by ALVAC and Ad5 remain unknown. However, a prominent difference between ALVAC and Ad5 vector is related to their intracellular locations for replication. After entry into target APCs, poxvirus replicates in cytoplasm [54], whereas adenovirus replicates in nucleus [55]. This may lead to engagement of different antigen presentation pathways (e.g. MHC class I vs. II) and therefore differential induction of CD8 vs. CD4 T-cell responses to these two vectors. Nevertheless, elicitation of vector-specific CD8 vs. CD4 responses by different vaccine vectors *in vivo* and the immune pathways involved remain less clear and should be further investigated.

Another interesting finding of this study is that unlike Ad5 vector, ALVAC-activated CD8 T cells can inhibit the proliferation and HIV infection of autologous vector-specific CD4 T cells (Fig 7 and Fig 8). Evidence presented in our study supports that the process may involve both lytic and non-lytic effects of CD8 T cells [46]. First, our trans-well experiments showed that CD8 T cells could still inhibit ALVAC-specific CD4 T cell proliferation (Fig 7C) and HIV susceptibility (Fig 8C) even in the absence of cell contact, indicating that soluble factors play a role in mediating the inhibitory effects of CD8 T cells. Indeed, we demonstrate that compared to Ad5 vector, ALVAC-activated CD8 T cells manifest a stronger Treg potential (CD25+FoxP3+) (Fig 7D) and produce higher levels of β-chemokines (Fig 8D), which may respectively inhibit ALVAC-specific CD4 T-cell proliferation and HIV susceptibility. Other than the non-lytic mechanisms, our data suggest that the cytotoxic effects of CD8 T cells may also play a role. We found that the presence of CD8 T cells in either whole PBMC or in trans-well culture (depleted CD8 T cells were added back) caused significant cytotoxic effect on total CD4 T cells (Fig 7E and 7F) as well as on ALVAC-specific CD4 T cells (Fig 8E). In support, we further demonstrate that compared to Ad5 vector, ALVAC-activated CD8 T cells manifest a stronger cytolytic and antiviral phenotype, expressing elevated levels of perforin, IFN-γ, and MIP-1β (Fig 9). Collectively, our observation that preferential induction of strong vector-specific CD8, but not CD4, T-cell proliferation by ALVAC as compared to Ad5 vector provides some new insights into our understanding of vaccine-induced immunity in HIV vaccination.

In summary, we here present strong evidence that CD4 T cells activated via different HIV vaccine vectors manifest distinct susceptibility to HIV infection, which is closely associated with their phenotypic and functional characteristics. Our findings suggest that future efforts should focus on candidate vaccine vectors that can maximize immunogenicity while minimizing potential HIV susceptibility, for example, by inducing low levels of vector-specific CD4 T cells with high HIV resistance. Future studies will seek to extend this analysis to other important HIV vaccine vectors and to further explicate the mechanism underlying differential HIV susceptibility of vector-specific CD4 T cells. Research that aims to understand how vector-specific CD8 T cells may exert anti-HIV activity and the immune pathways by which ALVAC stimulates strong vector-specific CD8 T-cell proliferation should also be of interest.

## Materials and methods

### Ethics statement and study participants

The study involves use of PBMC samples from two HIV vaccine clinical trials: RV144 (NCT00223080) (ALVAC-HIV prime/gp120 protein boost) and HVTN204 (NCT00125970) (DNA prime/rAd5 boost). De-identified, cryopreserved PBMC collected from vaccine responders of these two trials were used. All samples were analyzed anonymously and investigators of this study have no access to any subject identification information. The study was determined as non-human subject research and approved by the University of Texas Medical Branch’s IRB. Written informed consents were obtained from study participants.

### Cells, HIV, and viral vectors

PBMC were maintained at 37°C, 5% CO_2_ in RPMI medium (Invitrogen) supplemented with 10% human serum, 100 U/mL penicillin G, 100 U/mL streptomycin sulfate, and 1.17mM sodium glutamine. R5 (US1) and X4 (92/UG/029) HIV-1 (original stock from NIH) was used for *in vitro* infection of PBMC. HIV transmitted founder virus (TFV) strains (including AD17 clone) were a kind gift from Dr. Jason Kimata of Baylor College of Medicine. Empty ALVAC vector was obtained from Sanofi, and empty rAd5 vector was obtained from the Vaccine Research Center (VRC) of NIH.

### CFSE staining, vector stimulation, and HIV infection of PBMC

PBMC were CFSE labeled as described previously with slight modifications [20, 21, 23]. Thawed and washed PBMC at a concentration of 20 x 10^6^ PBMC/mL were stained in 1μM CFSE for 8 minutes at 25°C. Cells were then quenched with 2 mL of warm normal human serum for 5 minutes. Empty ALVAC or rAd5 vector corresponding to the original vaccine was used to re-stimulate CFSE-labeled PBMC (MOI of 3). Unstimulated PBMC were included as a control. Three days after stimulation, cells were exposed to pre-titrated R5 HIV, X4 HIV, or TFV HIV for *in vitro* infection. Three days after HIV exposure, HIV infection in CD4 T cells was analyzed by flow cytometry based on intracellular HIV p24 expression. For viral kinetics experiments, HIV infection rate was measured at 3 and 9 days post infection. In some experiments, anti-MIP-1α (5μg/mL; clone 93321; R&D Systems), anti-MIP-1β (5μg/mL; clone 24006; R&D Systems), and anti-RANTES (5 μg/mL; clone 21418; R&D Systems) were added to the cultures throughout the experiments to neutralize β-chemokines. In some experiments, anti-human IFNAR antibody (Abcam, final concentration: 5 μg/ml) was added to the cultures throughout the experiments to block type-I IFN signaling.

### CD8 T cells depletion, isolation and trans-well co-culture

In some experiments, CD8^+^ cells were depleted from PBMC using the EasySep Human CD8 Positive Selection Kit (Stem Cell Technologies, cat #17833) for comparison with whole PBMC. In the trans-well co-culture experiment, CD8 T cells were isolated from PBMC of RV144 vaccine recipients using the EasySep™ Human CD8+ T Cell Isolation Kit (StemCell Technologies) according to the manufacturer’s protocol after CFSE labeling. After CD8 T cell isolation, CD8 depleted PBMC and the corresponding whole PBMC were infected with ALVAC (MOI = 1), followed by HIV infection as describe above. In addition, isolated CD8 T cells were added back to the trans-well culture to explore mechanisms underlying CD8 T cell-mediated inhibition. Briefly, CD8-depleted PBMC were placed in the bottom chamber of the trans-well co-culture system, and the isolated autologous CD8 T cells were added back to the top chamber. The trans-well culture was also stimulated by ALVAC and infected with HIV as described above. HIV susceptibility and cellular phenotypes for different conditions (whole PBMC, CD8-depleted PBMC, CD8-depleted PBMC with added CD8 T cells in trans-well) were similarly measured by multi-color flow cytometry as described.

### Flow cytometric surface, intracellular cytokine and p24 staining and analysis

CFSE staining, vector stimulation and *in vitro* HIV infection of PBMC were conducted as described above. On day 6 after vector stimulation (3 days after HIV infection), cells were subjected to immune staining and flow cytometric analysis to examine the phenotypes and HIV susceptibility of vector-specific CD4 T cells. Cells were first stained with LIVE/DEAD fixable aqua dead cell stain (ThermoFisher Scientifc, cat #L34957) and antibodies to surface markers including CD3, CD4, CD8, CCR5, α4β7-APC (NIH AIDS Reagent Program), CCR7, PD-1, CD25 and CD45RO. Except α4β7, all surface antibodies were from BD Bioscience. Cells were then fixed, permeabilized (BD Biosciences cat #554722), and stained for HIV p24 (Beckman Coulter) for measuring HIV susceptibility of vector-specific CD4 T cells in PBMC (p24+ rate in CFSE-low CD4 T cells). In some experiments that also measured the expression of intracellular cytokines in vector-specific CD4 cells, cells were treated with phorbol 12-myristate 13-acetate (PMA) and ionomycin for 5 hours prior to staining in order to stimulate *de novo* cytokine production. After fixation and permeabilization, cells were also stained for intracellular cytokines IFN-γ, IL-2, IL-17, IL-21, (Biolegend), MIP-1β (BD Biosciences). In experiments that measured the antiviral and cytolytic profile of vector-specific CD8 T cells, anti-CD107a antibody (BD Biosciences) was added during cell stimulation. After fixation and permeabilization, cells were also intracellularly stained for perforin and Granzyme B (BD Bioscience). In experiments that measured regulatory T cells, cells were permeabilized using a FoxP3 Staining Buffer Set (eBioscience cat #00-5523-00) and stained for FoxP3 (Biolegend). Antibody capture compensation beads (BD Biosciences) stained with individual antibodies were prepared for compensation. Cell samples and compensation beads were acquired at LSR-II (BD). Flow cytometric data were analyzed using FlowJo Version 10 software (TreeStar).

### Cell sorting and real-time PCR for gene expression

Vaccine trial PBMC were CFSE stained and vector stimulated as described above. After 6 days of proliferation, cells were stained for CD3, CD4 and viability (Live/Dead Fixable Violet). The CFSE-low, CD3+CD4+ T cells were sorted from PBMC using FACSAria IIU (BD Biosciences). Total RNA was the sorted cells using Quick-RNA MicroPrep Kit (Zymo) according to the manufacturer’s protocol. Gene expression was quantified using iTaq Universal SYBR Green Supermix (Bio-Rad) and the CFX Connect Real-Time PCR Detection System (Bio-Rad) after reverse transcription from RNA into cDNA using iScript Reverse Transcription Supermix for RT-qPCR (Bio-Rad). Primer sequences for quantification of gene expression are shown in S1 Table. The relative quantity of gene expression was calculated using the 2−ΔΔCt method.

### Statistical analysis

Statistical analysis was performed using GraphPad Prism 6 (GraphPad, Inc.) Two-tailed, unpaired Student’s T tests were performed and a *p* value ≤ 0.05 considered significant. Ratio-paired T tests were performed where appropriate.

## Supporting information

S1 Fig Summary and verification of the *in vitro* HIV infection system. **(A)** Summary of the system. PBMCs from human individuals who were positive for CD4 responses to antigen of interest (e.g. to natural infections or vaccination) were CFSE-labeled and then stimulated with recall antigens (antigens from pathogens or vaccines) for ~3 days, followed by exposure to R5 or X4 HIV. Productive HIV infection in antigen (Ag)-specific CD4 T cells was determined based on flow cytometric analysis of intracellular p24 in CFSE-low, proliferating CD4 T cells. **(B)** Assessment of Ag specificity of the CFSE-low, expanded CD4 T cells. We here used CMV antigen as an example, since CMV-specific CD4 T cells manifest a polarized Th1 response with the majority of them producing one same cytokine (IFN-γ), making the assessment of Ag specificity more straightforward. Also, *in vivo* phenotypes of CMV-specific CD4 T cells have been well characterized and can used for comparison with those expanded *in vitro* in our system. Proliferating T cells were re-stimulated by the same recall antigen (CMV; APC-loaded) on day 6 after initial antigen stimulation. We confirmed that the CFSE-low, CD4 T cells were mostly antigen specific since >91% of them produced cytokine (IFN-γ) upon Ag re-stimulation. **(C)** *In vitro* expanded antigen-specific CD4 T cells closely resemble their *in vivo* phenotypes. CFSE-low, CMV-specific CD4 T cells were gated (top) for phenotypic analysis regarding memory differentiation (middle) and cytokine profile (bottom). *In vitro* proliferating CMV-specific cells were largely effector memory cells (CD27−CD45RO+) (81.8%), and a significant fraction of them were terminally differentiated (CD27−CD57+) (20.1%), consistent with their *in vivo* phenotypes. For cytokine expression, a majority of them co-expressed IFN-γ and MIP-1β (83.2%) but very little IL-2 (1.5%). Altogether, the *in vitro* proliferating Ag-specific CD4 T cells in our system well mirror their in vivo phenotypes.
(TIF)

S2 Fig HIV infection of CFSE-low vector-induced CD4 T cells at multiple time points after HIV exposure. RV144 (left) or HVTN204 (right) PBMC were CFSE-labeled, vector stimulated and HIV-infected as described above. Productive HIV infection in CFSE-low, vector-induced CD4 T cells was measured by flow cytometry at multiple time points (Day 3 and Day 9) after HIV exposure. Number in each panel shows intracellular p24+% in CFSE-low CD4 T cells.
(TIF)

S3 Fig Stimulation of T-cell proliferation by vectors in control PBMC and intracellular p24 staining in HIV uninfected CD4 T cells. **(A)** Pre-vaccine PBMC (left) and post-vaccine PBMC (right) from RV144 (top) and HVTN204 (bottom) vaccine recipients were CFSE-labeled, and respectively stimulated with ALVAC or Ad5 vector. CD3+ total T cells were gated and T-cell proliferation (CD8 and CD4) was analyzed on day 6 after stimulation by flow cytometry. **(B)** Post-vaccine PBMC from RV144 (top) and HVTN204 (bottom) were CFSE-labeled and respectively stimulated with ALVAC or Ad5 vector for 3 days, followed by HIV infection (R5; US-1) or not. 3 days after infection, CD3+CD8- T cells were gated and HIV infection in CFSE-low CD3+CD8- T cells was analyzed by flow cytometry based on intracellular p24 expression. Cells with no HIV infection were used to set up the gate for intracellular p24 staining (left panels).
(TIF)

S4 Fig HIV susceptibility of polyclonally stimulated CD4 T cells in PBMC. RV144 (left) and HVTN204 (right) PBMC were CFSE-labeled and then polyclonally stimulated with anti-CD3/CD28, followed by HIV infection (US-1) or not. HIV infection in proliferating CFSE-low CD4 T cells was measured by flow cytometry on day 6 as described above.
(TIF)

S5 Fig *In vitro* HIV susceptibility of vector-induced CD4 T cells to transmitted/founder virus HIV infection (TFV). HIV infection was conducted as described above, except that the transmitted/founder virus (TFV) (AD17 clone; virus prepared by Jason T. Kimata) was used for infection. Productive HIV infection in CFSE-low, vector-induced CD4 T cells in HVTN204 (left) or RV144 (right) PBMC was determined as described above.
(TIF)

S6 Fig *In vitro* HIV susceptibility of vaccine Env-specific CD4 T cells in PBMC of RV144 and HVTN204. PBMC of RV144 or HVTN204 HIV vaccine recipients were stained with CFSE and then re-stimulated with Env peptides for three days before being infected with CCR5-tropic (top) or CXCR4-tropic (bottom) HIV. HIV infection rate in Env-specific CD4 T cells was determined using flow cytometry to measure p24 expression 3 days post infection and expressed as the % p24+ CFSE-low CD4 T cells. Representative flow cytometry plots shown at left were gated on CD3+CD8- T cells.
(TIF)

S7 Fig Tfh, Treg and PD-1 analysis of vector-specific CD4 T cells. CFSE-labeled RV144 and HVTN204 PBMC were respectively stimulated with ALVAC or Ad5 as described for 6 days. Cells were analyzed for expression of different markers as indicated by flow cytometry. **(A)** Expression of Tfh cytokine IL-21 in CFSE-low CD4 T cells. Representative flow cytometry plots and cumulative results comparing the % IL-21+, CFSE-low CD4 T cells between ALVAC- and Ad5-specific CD4 T cells were shown. **(B)** Flow cytometric analysis of HIV infection (intracellular p24) in IL-21+ and IL-21- subsets of CFSE-low, Ad5-specific CD4 T cells. Numbers in the plots show % p24+, in IL-21+ (upper right quadrant) and IL-21- (upper left quadrant) subset of Ad5-specific CD4 T cells. **(C)** Expression of Treg markers (CD25 and FoxP3) in CFSE-low CD4 T cells. Representative flow cytometry plots and cumulative results comparing the % CD25+FoxP3+ CD4 T cells between ALVAC- and Ad5-specific CD4 T cells were shown. **(D)** PD-1 expression on vector-specific CD4 and CD8 T cells. Representative flow cytometry plots and cumulative results comparing the % PD-1+ between ALVAC- and Ad5-specific CD4 and CD8 T cells were shown. n.s.: non-significant.
(TIF)

S8 Fig Profile of vaccine Env-specific CD8 vs. CD4 T-cell proliferative response in RV144 and HVTN204 PBMC. PBMC were stained with CFSE and re-stimulated with Env peptides for 6 days. CD8 and CD4 T cell proliferation in stimulated PBMC was measured by flow cytometry. Live CD3+ T cells were gated for analysis.
(TIF)

S9 Fig No significant cell proliferation was detected in RV144 PBMC early at day 3 post ALVAC stimulation. RV144 PBMC were CFSE-labeled and stimulated with ALVAC for 3 days as described. Cell proliferation (CD3+ T cells and CD3- non-T cells) was measured by flow cytometry based on CFSE intensity.
(TIF)

S10 Fig Impact of CD8 depletion on expression of CCR5 and activation markers (CD25 and CD69) on CFSE-low, ALVAC-specific CD4 T cells. Three conditions of one RV144 PBMC (Whole PBMC, CD8-depleted PBMC, and CD8 addition back in trans-well) were CFSE-labeled and stimulated ALVAC as described. On day 6, CCR5 (top), CD25 (middle) and CD69 (bottom) expression on CFSE-low CD4 T cells was measured by flow cytometry.
(TIF)

S1 Table Real-time PCR primer sequences. (TIF)
