# Supplementary figures and images for: Distinct susceptibility of HIV vaccine vector-induced CD4 T cells to HIV infection
Source: PLoS Pathog. 2018 Feb 23;14(2):e1006888. doi: 10.1371/journal.ppat.1006888 (PMC5841825; doi:10.1371/journal.ppat.1006888)

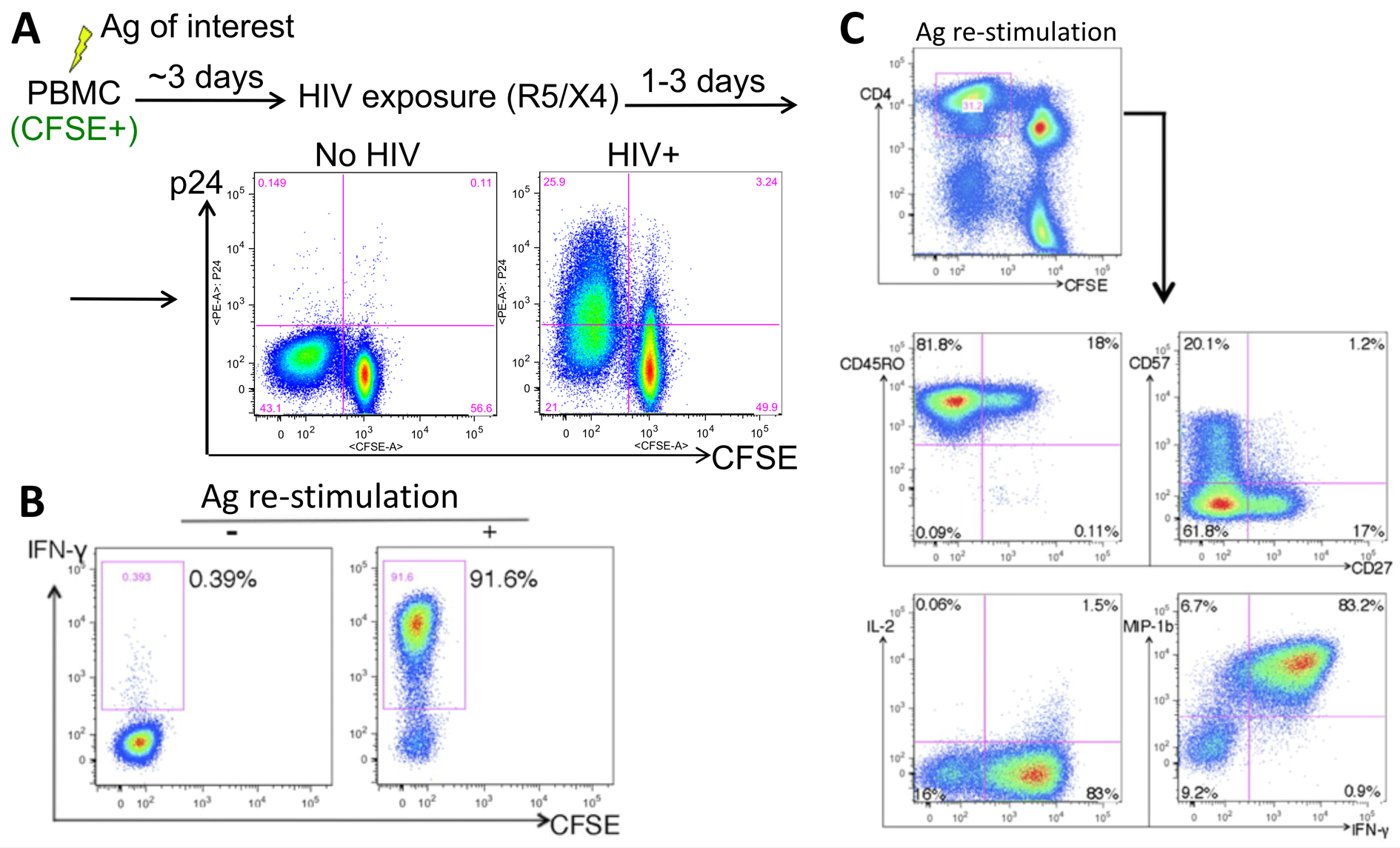

Supplement: S1 Fig — (A) Summary of the system. PBMCs from human individuals who were positive for CD4 responses to antigen of interest (e.g. to natural infections or vaccination) were CFSE-labeled and then stimulated with recall antigens (antigens from pathogens or vaccines) for ~3 days, followed by exposure to R5 or X4 HIV. Productive HIV infection in antigen (Ag)-specific CD4 T cells was determined based on flow cytometric analysis of intracellular p24 in CFSE-low, proliferating CD4 T cells. (B) Assessment of Ag specificity of the CFSE-low, expanded CD4 T cells. We here used CMV antigen as an example, since CMV-specific CD4 T cells manifest a polarized Th1 response with the majority of them producing one same cytokine (IFN-γ), making the assessment of Ag specificity more straightforward. Also, in vivo phenotypes of CMV-specific CD4 T cells have been well characterized and can used for comparison with those expanded in vitro in our system. Proliferating T cells were re-stimulated by the same recall antigen (CMV; APC-loaded) on day 6 after initial antigen stimulation. We confirmed that the CFSE-low, CD4 T cells were mostly antigen specific since >91% of them produced cytokine (IFN-γ) upon Ag re-stimulation. (C) In vitro expanded antigen-specific CD4 T cells closely resemble their in vivo phenotypes. CFSE-low, CMV-specific CD4 T cells were gated (top) for phenotypic analysis regarding memory differentiation (middle) and cytokine profile (bottom). In vitro proliferating CMV-specific cells were largely effector memory cells (CD27−CD45RO+) (81.8%), and a significant fraction of them were terminally differentiated (CD27−CD57+) (20.1%), consistent with their in vivo phenotypes. For cytokine expression, a majority of them co-expressed IFN-γ and MIP-1β (83.2%) but very little IL-2 (1.5%). Altogether, the in vitro proliferating Ag-specific CD4 T cells in our system well mirror their in vivo phenotypes. (TIF) [file ppat.1006888.s001.tif]

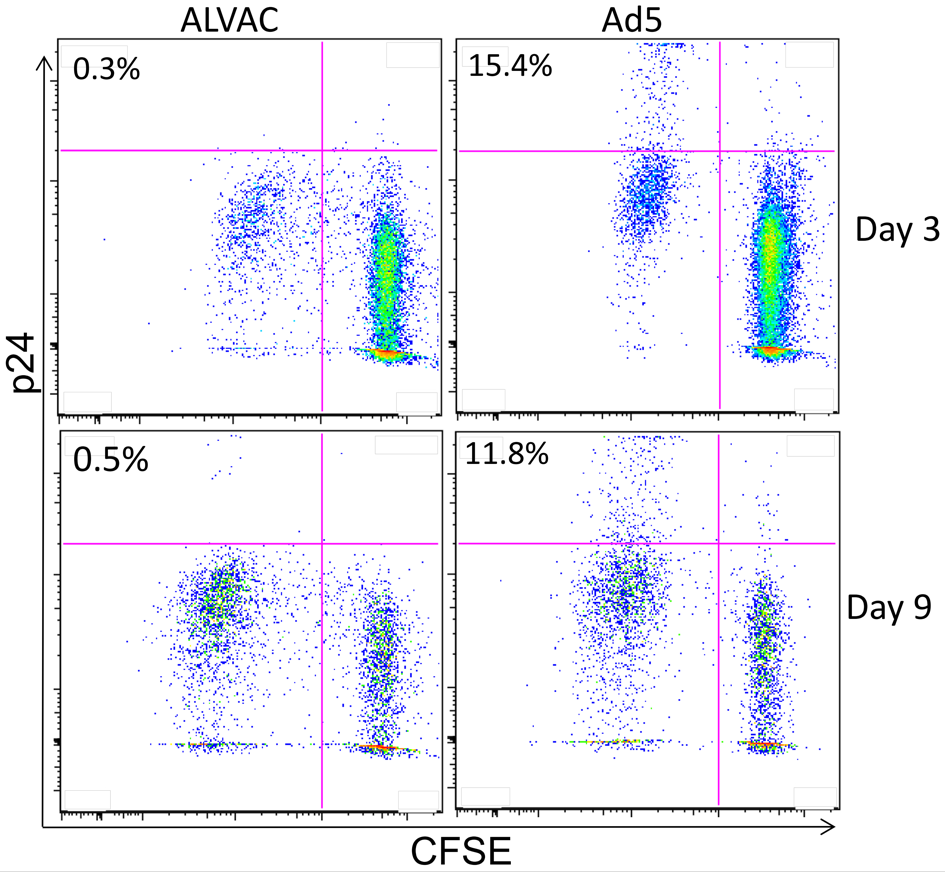

Supplement: S2 Fig — RV144 (left) or HVTN204 (right) PBMC were CFSE-labeled, vector stimulated and HIV-infected as described above. Productive HIV infection in CFSE-low, vector-induced CD4 T cells was measured by flow cytometry at multiple time points (Day 3 and Day 9) after HIV exposure. Number in each panel shows intracellular p24+% in CFSE-low CD4 T cells. (TIF) [file ppat.1006888.s002.tif]

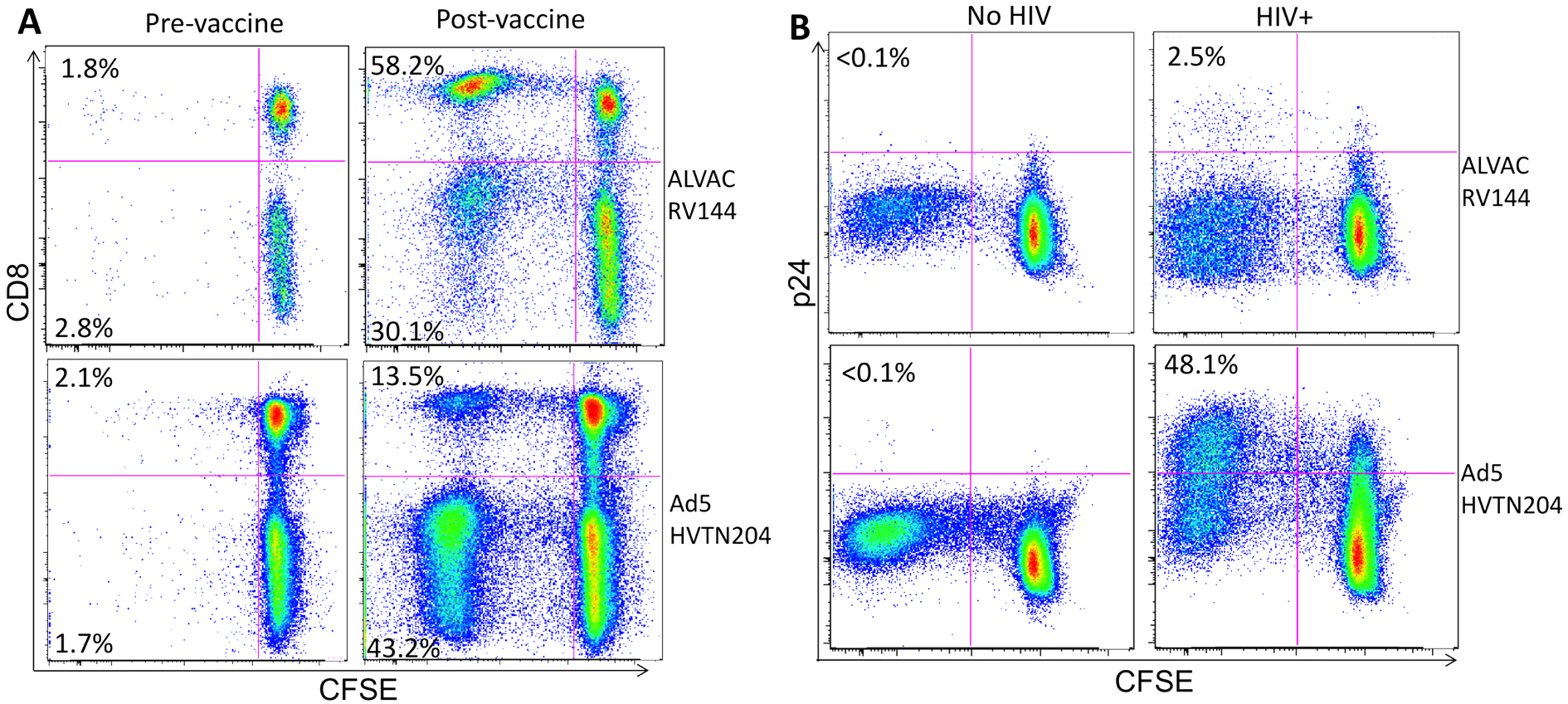

Supplement: S3 Fig — (A) Pre-vaccine PBMC (left) and post-vaccine PBMC (right) from RV144 (top) and HVTN204 (bottom) vaccine recipients were CFSE-labeled, and respectively stimulated with ALVAC or Ad5 vector. CD3+ total T cells were gated and T-cell proliferation (CD8 and CD4) was analyzed on day 6 after stimulation by flow cytometry. (B) Post-vaccine PBMC from RV144 (top) and HVTN204 (bottom) were CFSE-labeled and respectively stimulated with ALVAC or Ad5 vector for 3 days, followed by HIV infection (R5; US-1) or not. 3 days after infection, CD3+CD8- T cells were gated and HIV infection in CFSE-low CD3+CD8- T cells was analyzed by flow cytometry based on intracellular p24 expression. Cells with no HIV infection were used to set up the gate for intracellular p24 staining (left panels). (TIF) [file ppat.1006888.s003.tif]

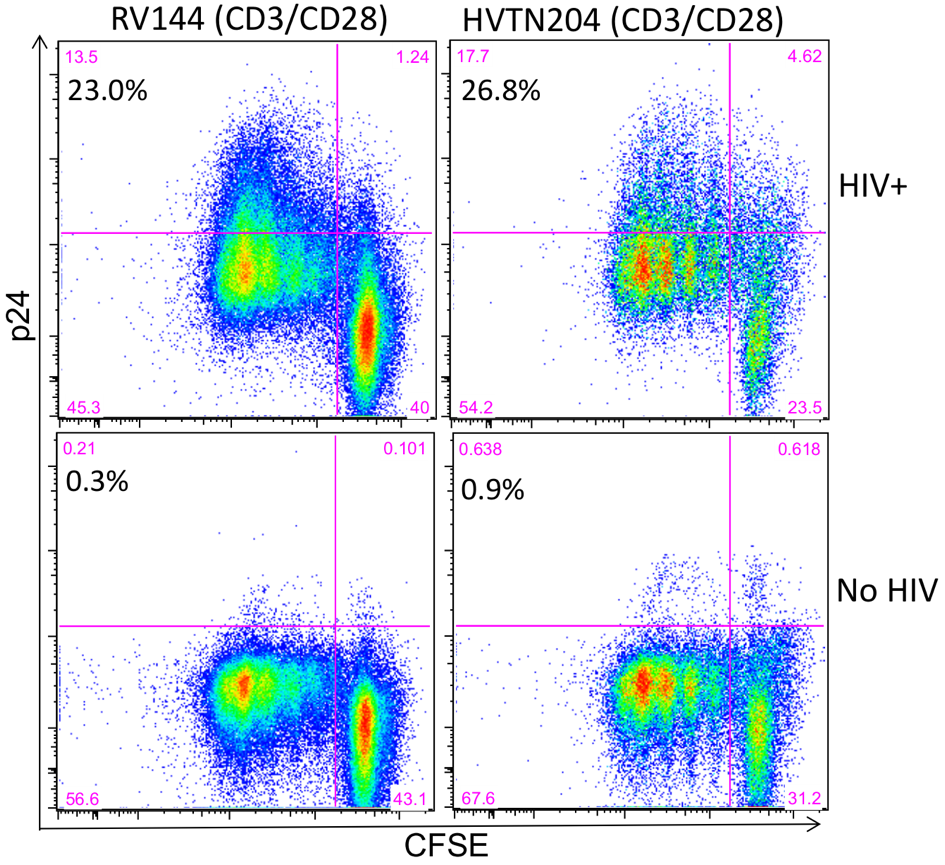

Supplement: S4 Fig — RV144 (left) and HVTN204 (right) PBMC were CFSE-labeled and then polyclonally stimulated with anti-CD3/CD28, followed by HIV infection (US-1) or not. HIV infection in proliferating CFSE-low CD4 T cells was measured by flow cytometry on day 6 as described above. (TIF) [file ppat.1006888.s004.tif]

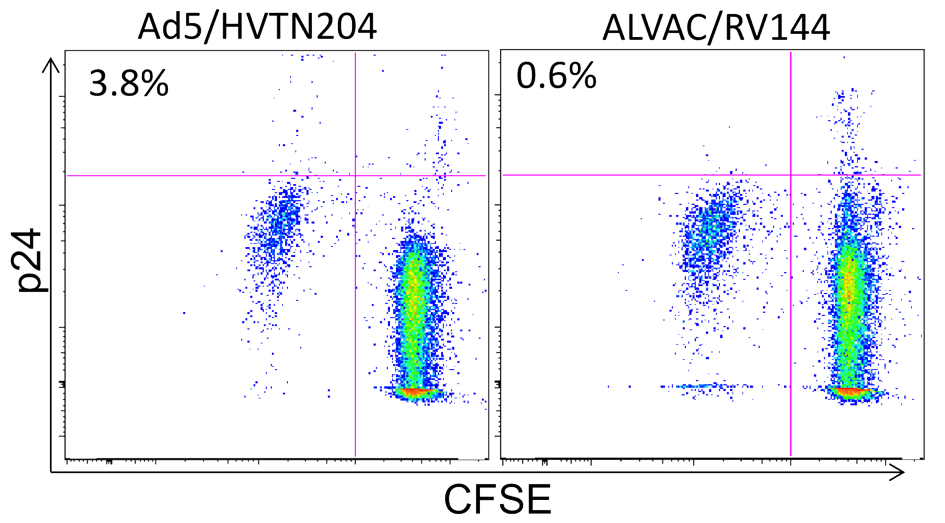

Supplement: S5 Fig — HIV infection was conducted as described above, except that the transmitted/founder virus (TFV) (AD17 clone; virus prepared by Jason T. Kimata) was used for infection. Productive HIV infection in CFSE-low, vector-induced CD4 T cells in HVTN204 (left) or RV144 (right) PBMC was determined as described above. (TIF) [file ppat.1006888.s005.tif]

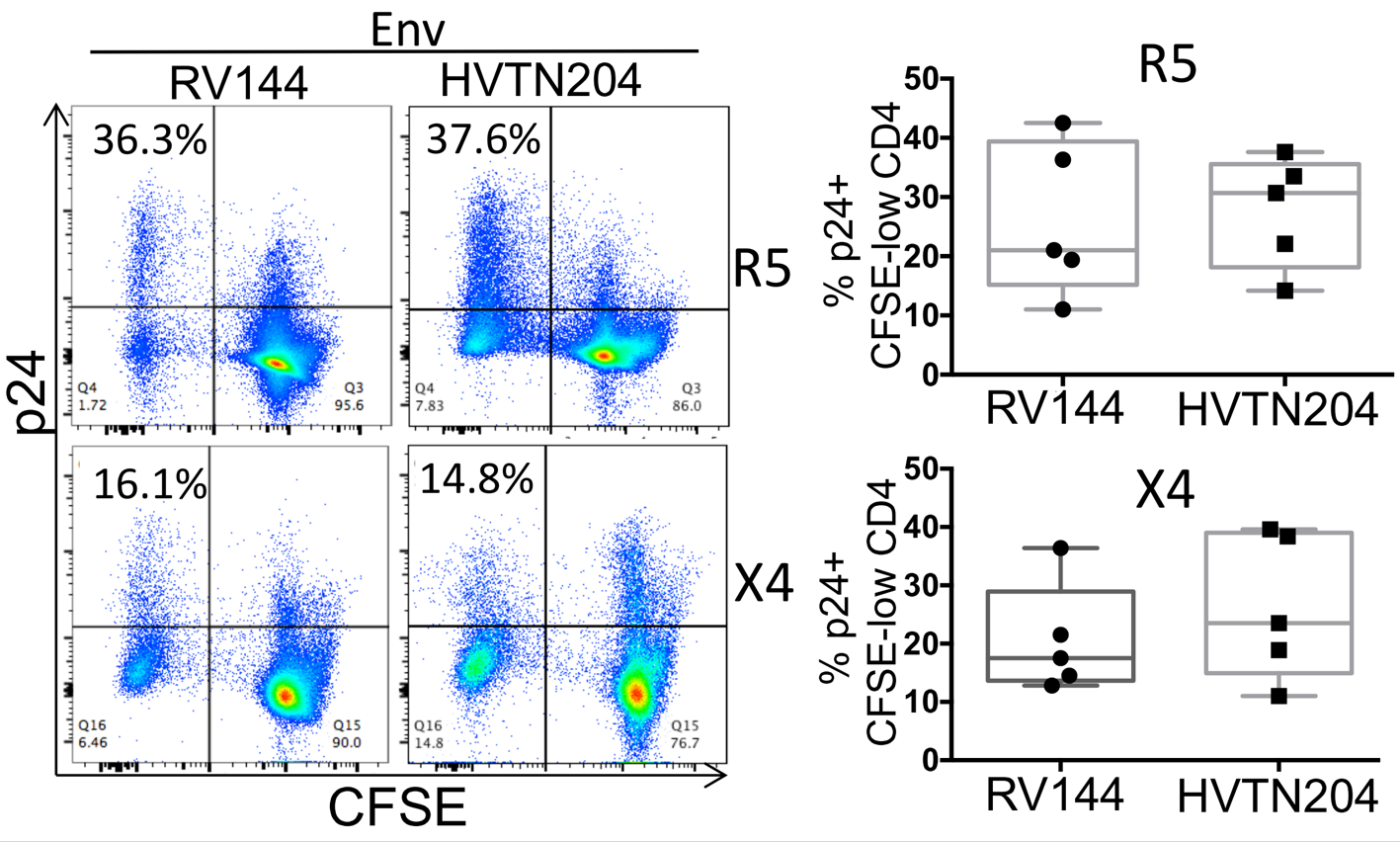

Supplement: S6 Fig — PBMC of RV144 or HVTN204 HIV vaccine recipients were stained with CFSE and then re-stimulated with Env peptides for three days before being infected with CCR5-tropic (top) or CXCR4-tropic (bottom) HIV. HIV infection rate in Env-specific CD4 T cells was determined using flow cytometry to measure p24 expression 3 days post infection and expressed as the % p24+ CFSE-low CD4 T cells. Representative flow cytometry plots shown at left were gated on CD3+CD8- T cells. (TIF) [file ppat.1006888.s006.tif]

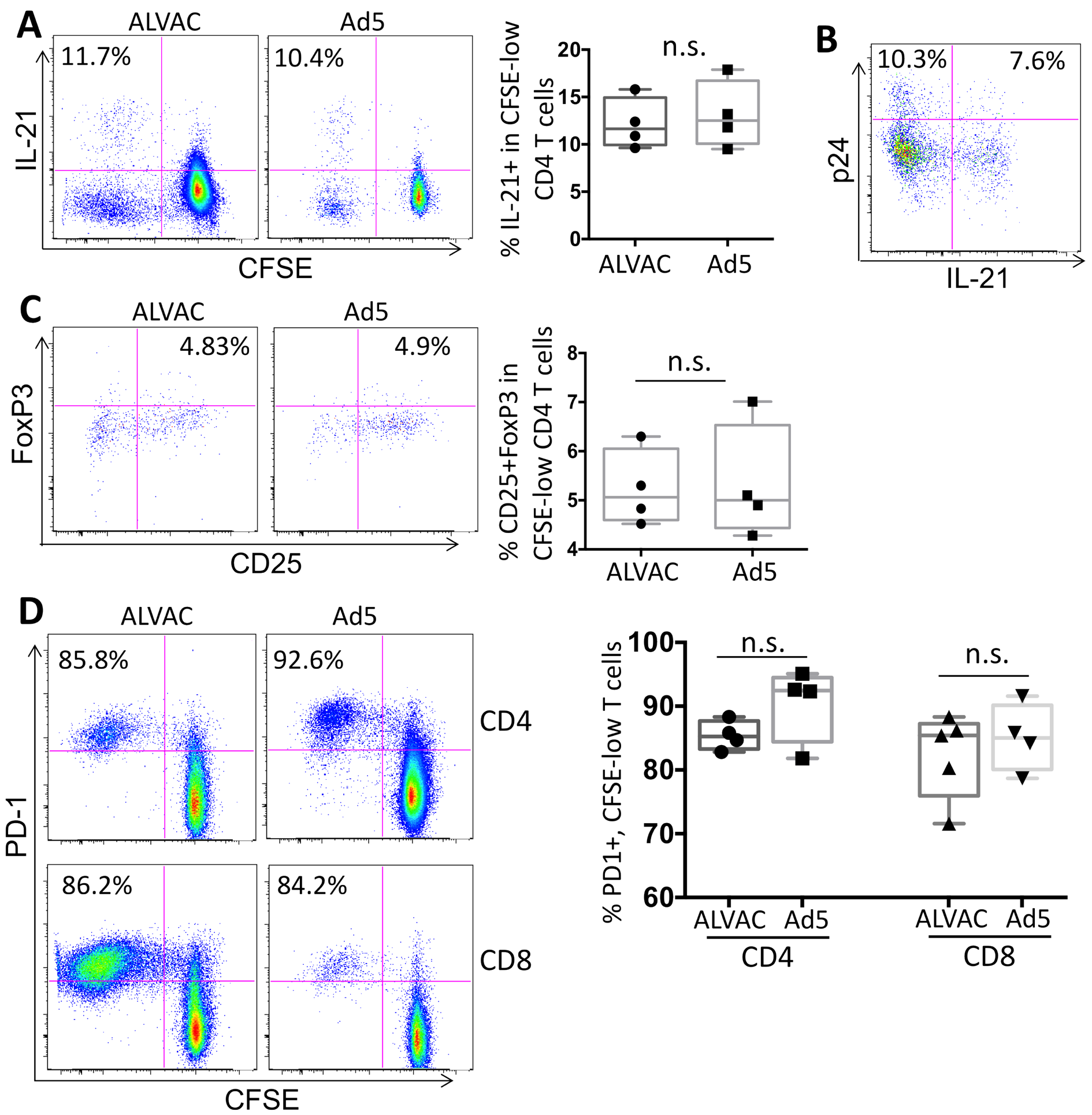

Supplement: S7 Fig — CFSE-labeled RV144 and HVTN204 PBMC were respectively stimulated with ALVAC or Ad5 as described for 6 days. Cells were analyzed for expression of different markers as indicated by flow cytometry. (A) Expression of Tfh cytokine IL-21 in CFSE-low CD4 T cells. Representative flow cytometry plots and cumulative results comparing the % IL-21+, CFSE-low CD4 T cells between ALVAC- and Ad5-specific CD4 T cells were shown. (B) Flow cytometric analysis of HIV infection (intracellular p24) in IL-21+ and IL-21- subsets of CFSE-low, Ad5-specific CD4 T cells. Numbers in the plots show % p24+, in IL-21+ (upper right quadrant) and IL-21- (upper left quadrant) subset of Ad5-specific CD4 T cells. (C) Expression of Treg markers (CD25 and FoxP3) in CFSE-low CD4 T cells. Representative flow cytometry plots and cumulative results comparing the % CD25+FoxP3+ CD4 T cells between ALVAC- and Ad5-specific CD4 T cells were shown. (D) PD-1 expression on vector-specific CD4 and CD8 T cells. Representative flow cytometry plots and cumulative results comparing the % PD-1+ between ALVAC- and Ad5-specific CD4 and CD8 T cells were shown. n.s.: non-significant. (TIF) [file ppat.1006888.s007.tif]

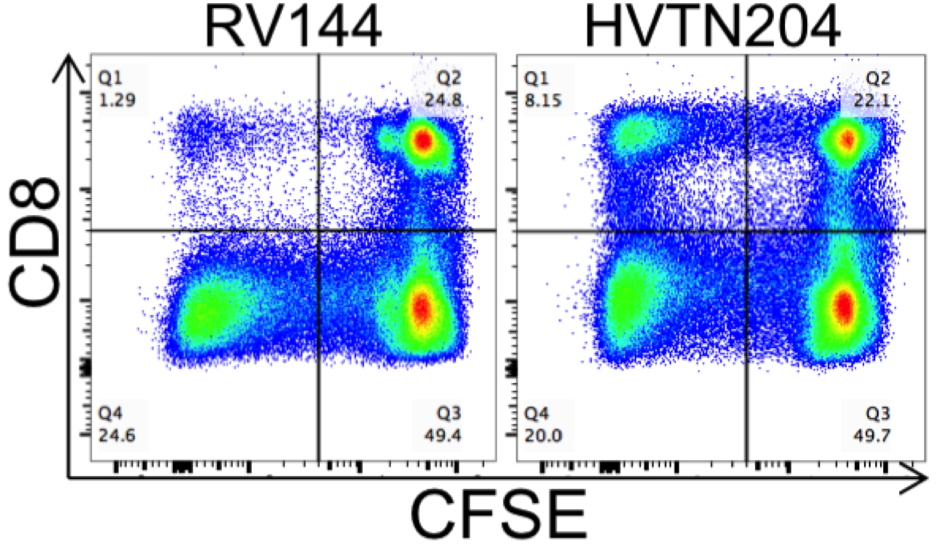

Supplement: S8 Fig — PBMC were stained with CFSE and re-stimulated with Env peptides for 6 days. CD8 and CD4 T cell proliferation in stimulated PBMC was measured by flow cytometry. Live CD3+ T cells were gated for analysis. (TIF) [file ppat.1006888.s008.tif]

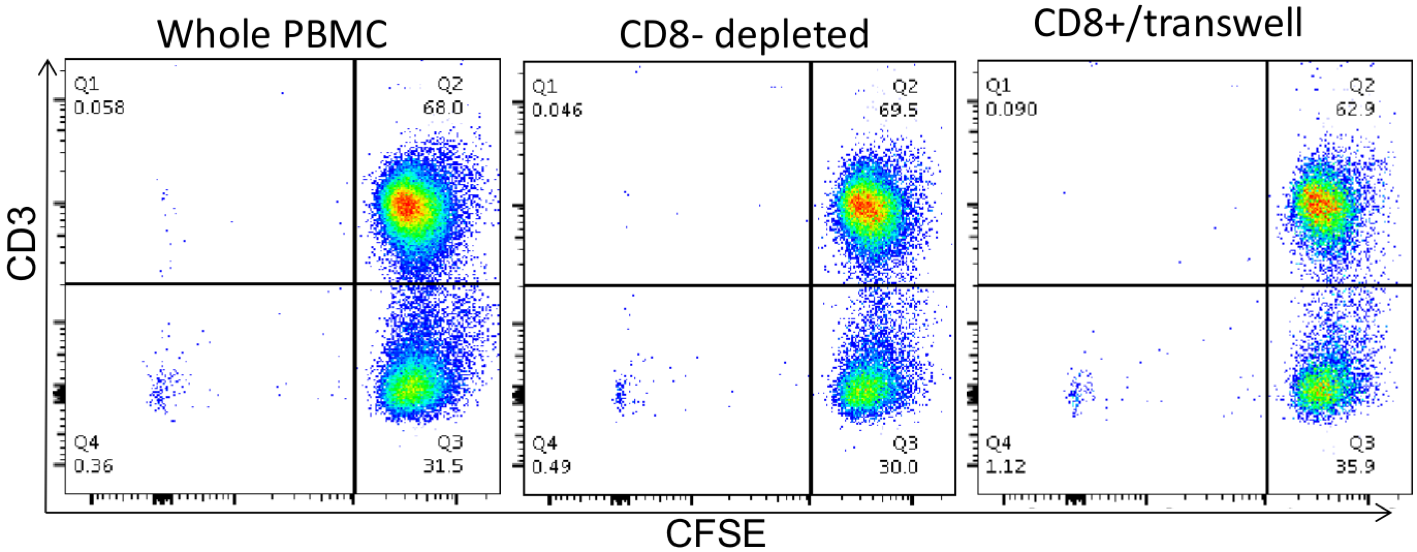

Supplement: S9 Fig — RV144 PBMC were CFSE-labeled and stimulated with ALVAC for 3 days as described. Cell proliferation (CD3+ T cells and CD3- non-T cells) was measured by flow cytometry based on CFSE intensity. (TIF) [file ppat.1006888.s009.tif]

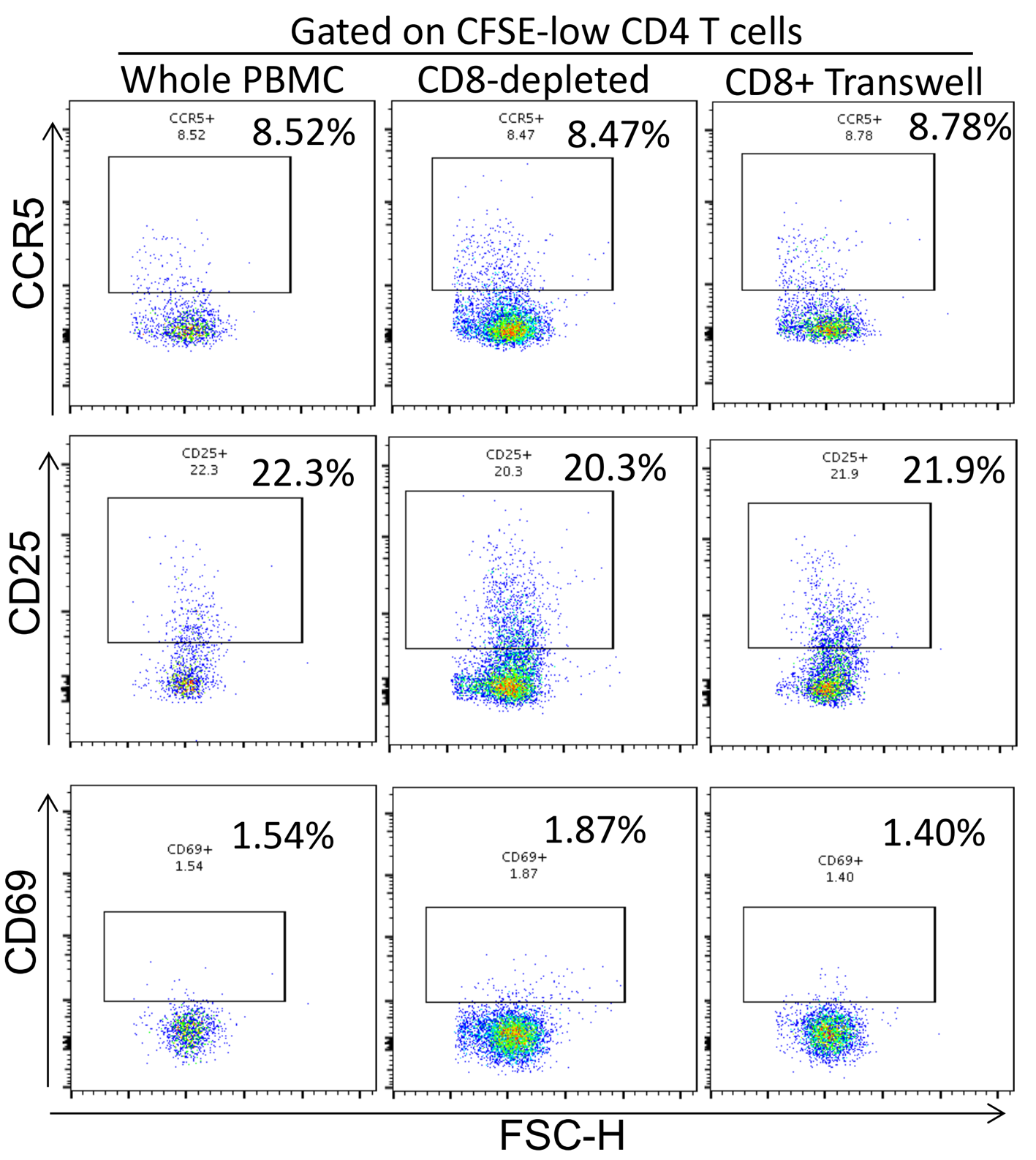

Supplement: S10 Fig — Three conditions of one RV144 PBMC (Whole PBMC, CD8-depleted PBMC, and CD8 addition back in trans-well) were CFSE-labeled and stimulated ALVAC as described. On day 6, CCR5 (top), CD25 (middle) and CD69 (bottom) expression on CFSE-low CD4 T cells was measured by flow cytometry. (TIF) [file ppat.1006888.s010.tif]

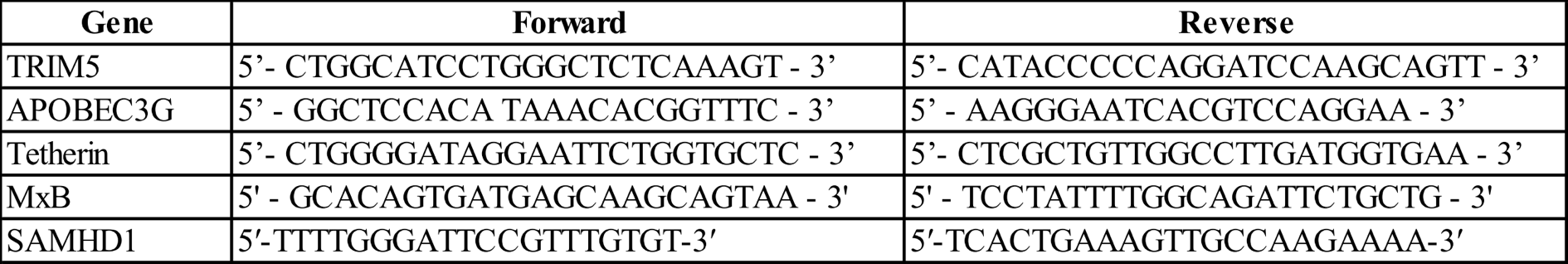

Supplement: S1 Table — (TIF) [file ppat.1006888.s011.tif]
